# Pharmacological Nephroprotection in Non-Diabetic Chronic Kidney Disease—Clinical Practice Position Statement of the Polish Society of Nephrology

**DOI:** 10.3390/jcm12165184

**Published:** 2023-08-09

**Authors:** Tomasz Stompór, Marcin Adamczak, Ilona Kurnatowska, Beata Naumnik, Michał Nowicki, Leszek Tylicki, Agata Winiarska, Magdalena Krajewska

**Affiliations:** 1 Department of Nephrology, Hypertension and Internal Medicine, University of Warmia and Mazury in Olsztyn, 10-516 Olsztyn, Poland; 2 Department of Nephrology, Transplantation and Internal Medicine, Medical University of Silesia, 40-027 Katowice, Poland; 3 Department of Internal Diseases and Transplant Nephrology, Medical University of Lodz, 90-419 Lodz, Poland; 4 Ist Department of Nephrology and Transplantation with Dialysis Unit, Medical University of Bialystok, Zurawia 14 St., 15-540 Bialystok, Poland; 5 Department of Nephrology, Hypertension and Kidney Transplantation, Central University Hospital, Medical University of Lodz, 92-213 Lodz, Poland; 6 Department of Nephrology, Transplantology and Internal Medicine, Medical University of Gdansk, 80-952 Gdansk, Poland; 7 Department of Nephrology and Transplantation Medicine, Wroclaw Medical University, 50-367 Wroclaw, Poland; magdalena.krajewska@umed.wroc.pl

**Keywords:** chronic kidney disease, blood pressure control, renin–angiotensin system, angiotensin-converting enzyme inhibitors, angiotensin II receptor blockers, sodium–glucose co-transporter type 2 inhibitors, metabolic acidosis, Fabry disease, autosomal-dominant polycystic kidney disease

## Abstract

Chronic kidney disease (CKD) is a modern epidemic worldwide. Introducing renin–angiotensin system (RAS) inhibitors (i.e., ACEi or ARB) not only as blood-pressure-lowering agents, but also as nephroprotective drugs with antiproteinuric potential was a milestone in the therapy of CKD. For decades, this treatment remained the only proven strategy to slow down CKD progression. This situation changed some years ago primarily due to the introduction of drugs designed to treat diabetes that turned into nephroprotective strategies not only in diabetic kidney disease, but also in CKD unrelated to diabetes. In addition, several drugs emerged that precisely target the pathogenetic mechanisms of particular kidney diseases. Finally, the role of metabolic acidosis in CKD progression (and not only the sequelae of CKD) came to light. In this review, we aim to comprehensively discuss all relevant therapies that slow down the progression of non-diabetic kidney disease, including the lowering of blood pressure, through the nephroprotective effects of ACEi/ARB and spironolactone independent from BP lowering, as well as the role of sodium–glucose co-transporter type 2 inhibitors, acidosis correction and disease-specific treatment strategies. We also briefly address the therapies that attempt to slow down the progression of CKD, which did not confirm this effect. We are convinced that our in-depth review with practical statements on multiple aspects of treatment offered to non-diabetic CKD fills the existing gap in the available literature. We believe that it may help clinicians who take care of CKD patients in their practice. Finally, we propose the strategy that should be implemented in most non-diabetic CKD patients to prevent disease progression.

## 1. Introduction

Chronic kidney disease (CKD) is defined as a reduced glomerular filtration rate (eGFR), increased urinary albumin excretion, or both, and is an increasing public health issue [1,2]. Its global prevalence ranges from 11 to 15%, with the majority of patients in stage 3, according to the National Kidney Foundation (NKF) [1]. In addition to the high prevalence of CKD, there is a significant burden of CKD-related complications, including cardiovascular disease, infections, and electrolyte imbalances. These complications lead to hospitalization, increased healthcare costs, and reduced quality of life in affected individuals. Moreover, all stages of CKD are associated with increased risks of premature mortality (mostly secondary to cardiovascular, cerebrovascular, and infectious causes) [3]. Globally, in 2017, 1.2 million people died from CKD [3].

CKD prevalence rates differ across regions. For example, the prevalence of CKD in the United States is approximately 14%; in Asia, it is estimated to be between 8% and 16%, while in Europe, about 12% [3]. For example, this problem affects 13% of the population over 16 years of age in the UK and one in 10 adults in Sweden. The burden of CKD is exceptionally high in certain countries, such as India, where it is estimated that over 230 million people have some stage of kidney disease [4]. Other countries with high rates of CKD include Pakistan, Bangladesh, and China [5,6].

In Poland, it is estimated that 4.2 million individuals suffered from CKD in 2020, with 90% being unaware of their condition [7]. At least 6500 people (about 170 per million inhabitants) lose their kidney function yearly in Poland, necessitating renal replacement therapy, which significantly burdens the healthcare system and generates enormous costs. After two years of the SARS-CoV-2 pandemic, this number is estimated to have increased to about 4.7 million patients.

These growing trends can be noticed in different countries. Based on an analysis of Medicare data in the United States, the prevalence of diagnosed CKD has steadily risen annually across all stages of CKD. Medicare spending on CKD and end-stage renal disease (ESRD) patients exceeded USD 120 billion in 2017 alone [8].

The globalization of the CKD problem is inextricably linked to the epidemic of other lifestyle diseases, such as obesity, diabetes mellitus, cardiovascular diseases, and hypertension [9]. Type 2 diabetes is a leading cause of ESRD worldwide. Despite higher risks for mortality and ESRD in diabetes, the relative chances of these outcomes by eGFR and UACR (urinary albumin/creatinine ratio) are more or less the same irrespective of the presence or absence of diabetes, emphasizing the importance of kidney disease as a predictor of clinical outcomes [10]. Cardiovascular morbidities, including hypertension, are significant risk factors for the development and progression of CKD, with evidence suggesting that nephroprotection is the critical management strategy for slowing down the progression of the disease and reducing the risk of cardiovascular events. Antihypertensive therapy and the inhibition of the renin–angiotensin–aldosterone (RAA) axis are established strategies for slowing the progression of CKD [1].

Additionally, sodium–glucose co-transporter type 2 inhibitors (SGLT2i) have recently emerged as promising new therapeutic options. These agents reduce the renal workload by decreasing sodium reabsorption, leading to a reduction in blood pressure and albuminuria, which are both associated with CKD progression. Nowadays, it is evident that the combination of RAA axis inhibitors and SGLT2i have additive and synergistic effects in reducing cardiovascular risk in CKD patients. Optimal blood pressure control and the correction of metabolic acidosis are crucial for preserving kidney function.

In addition to these general strategies, other diseases/diagnosis-specific nephroprotection therapies are essential in conditions such as autosomal-dominant polycystic kidney disease (ADPKD) and Fabry disease. Nowadays, disease-modifying treatment, e.g., an arginine vasopressin V2 receptor antagonist or enzyme replacement therapy with recombinant alpha-galactosidase A, has also been available in daily nephrological practice.

In the face of new nephroprotective therapies, the asymptomatic course of the early stages of CKD requires systemic actions towards mandatory preventive examinations, especially in risk groups. The benefits of this go beyond treatment, which can slow or even stop the progression of CKD. This could help to reduce cardiovascular risk and healthcare costs.

To address the challenges of CKD management, it is essential to update and widely disseminate evidence-based recommendations for the diagnosis and treatment of CKD. Implementing these recommendations in clinical practice can help improve outcomes in CKD patients, reduce the burden of cardiovascular disease, and slow the progression of CKD. Faced with these objectives, the Polish Society of Nephrology has prepared a position statement for nephroprotection in non-diabetic CKD patients. The guidelines aim to provide optimal nephroprotection use and to emphasize the importance of the early detection and management of CKD. They will help clinicians make treatment decisions and ensure that patients receive the best care to prevent or delay the progression to ESRD. Although the precise definition of pharmacological nephroprotection is not universally accepted, in our opinion, these include the pharmacological interventions aimed at the preservation of or increase (in the presence of renal reserve) in the glomerular filtration rate and at the attenuation of other indices of renal damage (for example, albuminuria/proteinuria).

## 2. Antihypertensive Therapy

Statement 2.1. We suggest that adults with CKD and high blood pressure (BP) should be treated at a target office BP like in the general population—at least 130–139/70–79 mmHg (primary goal) and perhaps lower (120–129/70–79 mmHg) in most CKD patients (especially young people and/or those with proteinuria) [expert opinion].

Statement 2.2. Antihypertensive drugs should be started in most CKD patients with hypertension without unnecessary delay, together with lifestyle modifications [expert opinion].

Statement 2.3. We suggest using renin–angiotensin system inhibitors (RASi) (angiotensin-converting enzyme inhibitor (ACEi) or angiotensin II receptor blocker (ARB)) as a first line of antihypertensive therapy in people with high BP and CKD [expert opinion], and recommend ACEi or ARB in those with increased albuminuria (A2 (2C) and A3 (1B)). We suggest monitoring serum potassium and creatinine concentrations during ACEi or ARB therapy [expert opinion; see recommendation 3.1].

Statement 2.4. Combined antihypertensive therapy should be used in most hypertensive CKD patients. We suggest adding a dihydropyridine calcium channel blocker (CCB) and/or a diuretic (1B). In patients with eGFR > 30 mL/min/1.73 m^2^, thiazide or thiazide-like diuretics should be used; in patients with eGFR ≤ 30 mL/min/1.73 m^2^, chlorthalidone or loop diuretics should be used (1B). We suggest monitoring serum sodium and potassium concentrations in patients treated with thiazide diuretics (including hydrochlorothiazide), thiazide-like diuretics (including indapamide and chlortalidone), and loop diuretics (including furosemide and torsemide) [expert opinion].

Statement 2.5. We suggest adding steroid mineralocorticoid antagonist (MRA)—spironolactone in patients with resistant hypertension and no tendency to hyperkalemia (i.e., serum potassium concentration ≤ 4.5 mmol/L). In patients treated with spironolactone, serum potassium concentrations should be monitored (2D). In patients with hyperkaliemia or other contraindications to spironolactone, other antihypertensive drugs should be added to achieve the target BP: doxazosin, post-synaptic α1-receptor antagonists, clonidine, central presynaptic α1-receptor agonists, moxonidin, I1-imidazoline receptor agonist, or β-adrenergic antagonist (in patients without any competing indications, nebivolol or carvedilol should be preferred) [expert opinion].

Comment on Statement 2.1

Arterial hypertension prevalence increases with declining kidney function. In CKD stage 2, about one-third of patients are hypertensive, and when eGFR is below 60 mL/min/1.73 m^2^, the prevalence of hypertension exceeds >80% and reaches almost 100% in advanced CKD (stages 4 and 5 not dialyzed) [11]. Hypertension is an important determinant of CKD progression and a significant risk factor for cardiovascular (CV) events, the leading complication in this population [12,13]. Therefore, the management of high BP is a major task in CKD patients, with two primary objectives: the prevention of CV events and protection against CKD progression. The question of BP targets in patients with CKD remains open without convincing scientific evidence. The study that hoped to solve the dilemma of target BP values in the general population was a randomized controlled trial (RCT) named the Systolic Blood Pressure Intervention Trial (SPRINT) [14]. This study showed that among adults with hypertension, but without diabetes, lowering systolic blood pressure to a target goal of less than 120 mmHg, as compared with the standard goal of less than 140 mmHg, resulted in significantly lower rates of fatal and nonfatal CV events and death from any cause [14]. The SPRINT trial randomized 9361 nondiabetic individuals, over 50 years of age, with at least one CV disease (CVD) risk factor, to intensive (systolic BP < 120 mmHg) and standard (SBP < 140 mmHg) BP target arms. The study was terminated early because of substantial CVD and mortality benefits observed in the intensive BP reduction arm [15]. However, this study had several limitations, especially regarding the CKD population. The analysis of the CKD subgroup in that study showed a 28% relative risk reduction (RRR) of all-cause death, but no risk reduction (RR) in the composite primary CVD outcome or the composite kidney outcome (reduction in eGFR of ≥50% from baseline or ESKD). The risk reduction (−18%) of the primary CV outcome in the CKD subgroup was less pronounced than in the population without CKD (−30%). Furthermore, there was an increased risk of acute kidney injury (AKI) in the intensive BP control arm. There was a more rapid decline in eGFR over the first six months in the intensive BP control group, which was sustained beyond the sixth month of follow-up [6]. In patients with eGFR < 45 mL/min/1.73 m^2^, there was no reduction in CV risk in the intensive BP control group compared with the standard BP group (hazard ratio (HR), 0.92; 95% CI (confidence interval), 0.62–1.38), whereas the significant risk of AKI (HR, 1.73; 95% CI, 1.12–2.66) was observed [16].

Moreover, it is essential to note that the SPRINT study excluded individuals < 50 years of age, those with diabetes or proteinuria ≥ 1 g/24 h, autosomal-dominant polycystic kidney disease, glomerulonephritis treated with or likely to be treated with immunosuppressive therapy, and those with eGFR < 20 mL/min/1.73 m^2^. The mean baseline eGFR in SPRINT was 48 mL/min/1.73 m^2^, and the trial included only a few patients with CKD stage 4. Therefore, the study excluded patients with advanced CKD and its most important causes. Furthermore, it should be noted that in the SPRINT study, BP was measured using methods not widely adopted in everyday clinical practice, i.e., unattended automated office blood pressure measurement (AOBPM). Briefly, in unattended AOBPM, BP is measured in patients who stay in a separate room without the presence of other persons, including medical staff, using a preprogrammed blood pressure monitor. Unattended AOBPM requires significant resources, including trained staff, additional clinic space, additional nurses’ time, and ensuring the use of preprogrammed BP devices. Therefore, it would require effort to implement the mentioned BP assessment into routine clinical practice and, in our opinion, would not be widely implemented. The results of AOBPM are lower than regular office BP measurements [17]. Therefore, the implementation of SPRINT’s findings (SBP target < 120 mmHg from AOBPM measurements) in office BP measurements, the most frequently used in outpatient clinics, increases the risk of postural hypotension, falls, fractures, AKI, stroke, and a rapid decline in eGFR, mainly in those with renovascular disease [18].

Moreover, with SBP < 120 mmHg in patients with CKD, there is also a risk of excessive lowering of diastolic blood pressure (DBP), especially in older patients who often have low DBP and high pulse pressure because of advanced atherosclerosis and arterial stiffness [19]. Many studies have demonstrated that DBP lower than 70 mmHg is associated with a higher risk of CVD, recurrent CV events, and stroke (compared with DBP between 71 and 89 mmHg) [20,21]. To date, pivotal clinical trials designed to test the impact of BP lowering on the progression of kidney failure are lacking, or the data remain insufficient. A meta-analysis of 11 RCTs comparing lower versus higher BP goals found that intensive BP reduction in patients with eGFR < 60 mL/min/1.73 m^2^ with or without proteinuria was associated with reducing the risk of kidney failure (RR 0.76, 95% CI 0.64–0.89), but not in those without proteinuria (RR 1.03, 95% CI 0.83–1.25; *p*-value for subgroup heterogeneity = 0.03) [22,23]. The REIN-2 study showed no benefit of intensified BP control (higher DBP target of <90 mmHg vs. lower BP target of <130/80 mmHg) by adding felodipine to baseline ramipril therapy in proteinuric patients (mean eGFR 35 mL/min/1.73 m^2^ and proteinuria approximately 3 g/24 h) without diabetes [24]. In a recently published meta-analysis, the authors pooled individual-level data from seven trials: Modification of Diet in Renal Disease (MDRD), African American Study of Kidney Disease and Hypertension (AASK), Action to Control Cardiovascular Risks in Diabetes Study (ACCORD), SPRINT, Secondary Prevention of Small Subcortical Strokes Trial (SPS3), Effect of Strict Blood Pressure Control and Angiotensin-Converting Enzyme Inhibition on the Progression of Chronic Renal Failure in Pediatric Patients (ESCAPE), and Ramipril Efficacy in Nephropathy-2 (REIN-2). They showed that intensive (versus usual) BP control was associated with a lower risk of kidney outcome in unadjusted analyses. However, in the intention-to-treat analysis, intensive BP control was associated with a 20% lower risk of the renal replacement therapy initiation in those with CKD stages 4–5, but not CKD stage 3. When the analysis was limited to trials that only included adult patients (n = 5157), the authors found that those who achieved an SBP of <120 mmHg or an SBP of 120–140 mmHg had lower odds of developing the kidney outcome (odds ratio (OR) 0.29; 95% CI, 0.23 to 0.37 and OR 0.43; 95% CI, 0.33 to 0.57, respectively) compared with patients with SBP ≥ 140 mmHg in unadjusted analysis. The findings were slightly attenuated, but still statistically significant in adjusted analysis (OR 0.43; 95% CI, 0.33 to 0.57 and OR 0.58; 95% CI, 0.48 to 0.71) for the kidney outcome. There was no interaction between intensive BP control and the severity of albuminuria for kidney outcomes [25]. Considering the above, the evidence on lowering BP to specific targets on kidney failure progression remains inconclusive. Considering this, we suggest using the same target office BP in patients with CKD and high blood pressure as in the general population.

Comment on Statement 2.2

Lifestyle changes are widely recommended initial steps for BP control, even among CKD patients. When necessary, dietary interventions should also be implemented. CKD patients usually present a salt-sensitive BP phenotype, which contributes to increased CV risk and is associated with the faster progression of CKD [26,27]. In a small randomized study, the reduction in SBP by an additional 7% was observed following low-sodium diet implementation added to ACEi in non-diabetic CKD patients with hypertension. It was significantly larger than that achieved by adding ARB to ACEi (SBP reduction of an additional 2%). Moreover, this study showed a lowering of proteinuria by a low-sodium diet added to baseline ACEi [28]. Several studies and meta-analyses have suggested that lowering dietary sodium intake delays kidney disease progression [29,30]. Moderate-intensity physical activity for a cumulative duration of at least 150 min per week or to a level compatible with patient cardiovascular and physical tolerance is also widely recommended, since it has been observed that a dose–response relationship exists between increasing physical activity and decreasing the risk of mortality in CKD patients [31]. Cessation of smoking, weight control, and the moderation of alcohol consumption should be proposed in all CKD patients [31]. Still, data on the risks or benefits of these interventions on BP or clinical endpoints, specifically in CKD populations, are insufficient and do not allow for strict recommendations. Because nonpharmacologic interventions alone are inadequate in controlling hypertension and resistant hypertension is highly prevalent in this CKD population [32], antihypertensives should be used in most CKD patients without unnecessary delay.

Comment on Statement 2.3

The RASi are preferred agents in managing hypertension in CKD patients. The ACEi are effective in lowering BP. It has been shown that the mean decrease in SBP and DBP in nondiabetic CKD who received ACEi equaled 4.9 and 1.2 mmHg, respectively [33]. In the Angiotensin-Converting Enzyme Inhibitors and Kidney Protection (AIPRI) trial comparing benazepril to placebo in patients with CKD, mostly without diabetes, a DBP reduction of 3.5 to 5.0 mmHg was noted in the benazepril group. In contrast, it increased by 0.2 to 1.5 mmHg in patients receiving a placebo. In parallel, the mean SBP decreased by 4.5 to 8.0 mmHg in the benazepril group and increased by 1.0 to 3.7 mmHg in the placebo group [34]. Likewise, the meta-analysis of 24 studies involving both diabetic and non-diabetic CKD patients with hypertension showed that monotherapy with an ARB for >1 year significantly decreased SBP (mean difference (MD): −14.84 mmHg; 95% CI, −17.82 to −11.85; *p* < 0.01), DBP (MD −10.27 mmHg; 95% CI, −12.26 to −8.27; *p* < 0.01) and proteinuria (MD: −0.90 g/L; 95% CI, −1.22 to −0.59; *p* < 0.01) without any adverse impact on eGFR [35]. It must be noted that using RASi in hypertensive CKD patients is based mainly on their efficacy in reducing proteinuria or inhibiting kidney disease progression and/or reducing CV, which is considered at least partially independent of the BP-lowering effect.

The Heart Outcomes Prevention Evaluation (HOPE) study, one of the RASi trials that included CKD patients, in a prespecified subgroup analysis of those with CKD and normal or moderately increased albuminuria (creatinine clearance < 65 mL/min, estimated by the Cockcroft–Gault formula; mean follow-up 4.5 years), found that ACEi reduced the risk for all-cause mortality by 20% versus placebo (HR: 0.80; 95% CI: 0.67–0.96), myocardial infarction by 26% (HR: 0.74; 95% CI: 0.61–0.91), and stroke by 31% (HR: 0.69; 95% CI: 0.49–0.90) [36]. Several other subgroup analyses of individuals with impaired kidney function (eGFR < 60 mL/min/1.73 m^2^) showed a reduced risk of CV endpoints and all-cause death for ACEi vs. placebo [37,38,39].

Xie X et al., in the metanalysis of 119 controlled trials (n = 64,768), showed that both ACEi and ARBs used in CKD patients reduce the risk for kidney failure and CV events. Still, ACEi also reduced the risk for all-cause mortality and was possibly superior to ARBs for preventing kidney failure, CV death, and all-cause mortality in patients with CKD. ACEi could be the first-choice treatment in this population [40]. However, the Renoprotection of Optimal Antiproteinuric Doses (ROAD) study, which directly compared benazepril (ACEi) to losartan (ARB) in 360 patients with CKD without diabetes and mean proteinuria ranging between 1.4 and 2.0 g/24 h did not show differences in BP control, kidney outcomes, or CV complications between the two drug classes [41]. The role of RASi in kidney protection in CKD patients is discussed in the comments on statements 3.1 and 3.2. It should be kept in mind that RAS blockade in CKD may cause hyperkalemia or kidney function deterioration in some patients (especially those with bilateral renal artery stenosis or dehydration). Therefore, monitoring serum potassium and creatinine concentrations during ACEi or ARB therapy is mandatory (though the exact frequency of such testing cannot be recommended).

Comment on Statement 2.4

As documented in many studies, most patients with CKD require more than one medication to control BP [42,43,44]. Therefore, adding a dihydropyridine calcium channel blocker (CCB), diuretic, or both to ACEi/ARB therapy is a widely accepted treatment strategy [45]. CCBs are potent vasodilators that efficaciously lower BP in hypertensive patients with CKD. However, their ability to protect kidney function has long been questioned [44]. In the AASK trial (participants: hypertensive kidney disease patients with eGFR 20–65 mL/min/1.73 m^2^), metoprolol and amlodipine did not differ significantly from ramipril in terms of new CV event incidence. Still, they were inferior to ramipril in renal endpoints [44]. In many studies, the CCBs were repeatedly shown to be less effective than RASi in delaying kidney disease progression, despite a comparable reduction in BP [44,46]. The Avoiding Cardiovascular Events Through Combination Therapy in Patients Living With Systolic Hypertension (ACCOMPLISH) trial, in which only a certain proportion of patients suffered from CKD (with mean eGFR 45 mL/min/1.73 m^2^), compared the benefits of combining an ACEi + amlodipine versus ACEi + hydrochlorothiazide (HCTZ) in high-risk hypertensive patients and found a lower risk of CKD progression and reduced incidence of CV events when the ACEi was combined with amlodipine (as compared to ACEi + HCTZ) [47,48]. The recently published meta-analysis of 16 randomized controlled trials that included hypertensive patients with CKD treated with different BP-lowering regimens showed the most significant reduction of SBP and DBP with the ARB + CCB dual regimen over ACEi monotherapy (standardized mean difference (SMD) 17.60 for SBP and 9.40 for DBP), ACEi + CCB regimen (SMD 12.90 for SBP and 9.90 for DBP), and ARB monotherapy (SMD 13.20 for SBP and 5.00 for DBP) [49]. Consequently, combining CCB and a RASi is a good option as a first-line combination therapy for managing hypertension in CKD. Although the RASi and CCB dual regimen is the first choice, the possibility of effectively controlling BP, especially in advanced CKD without a diuretic, is low. Diuretics are also frequently needed due to the high prevalence of fluid overload and the sodium sensitivity of hypertension.

The pathophysiology of hypertension in renal insufficiency involves an expansion of the extracellular fluid because of a decreased capacity of the kidneys to excrete sodium [50]. For these reasons, diuretics are widely used to treat hypertension in patients with CKD and, even more so, in chronic kidney failure. In this setting, loop diuretics are the drugs of choice because they can increase fractional sodium excretion by 20% and are efficient regardless of the degree of the eGFR reduction [51]. Loop diuretics are recommended when eGFR falls below 30 mL/min/1.73 m^2^, and their doses require a stepwise increase along with kidney failure deterioration. The general concept that thiazide and thiazide-like diuretics are ineffective in advanced CKD has been challenged, as many small studies have demonstrated that thiazide diuretics can lower BP even in advanced CKD [52,53]. In one of them, furosemide (60 mg) and hydrochlorothiazide (25 mg) were compared. Initially, the two diuretics were given as single agents for three months. Then, both diuretics were combined for another three months in 23 patients with hypertension and CKD stages 4 or 5. HCTZ was as effective as furosemide in reducing BP, and combining thiazide with the loop diuretic had a synergistic effect [54]. A recent study by Agarwal et al. evaluated the efficacy of chlorthalidone in patients with CKD stage 4 and poorly controlled hypertension. Chlorthalidone therapy improved BP control (reduction of the 24 h ambulatory SBP: −10.5 mmHg and DBP −3.9 mmHg in the chlortalidone group was noted) at 12 weeks and reduced proteinuria compared to placebo [54]. It should be underlined that in the chlorthalidone group, the reduction in the eGFR was more pronounced than in the placebo group over 12 weeks. Still, two weeks after discontinuing the assigned trial regimen, the eGFR was similar in the two groups. As with loop diuretics, higher thiazide doses are necessary to achieve a therapeutic effect in CKD because these drugs act on the luminal side of the tubular epithelium. With reduced tubular mass in CKD, less medication is secreted into the tubular lumen [55].

A cross-sectional analysis showed that approximately one in five patients receiving a thiazide or thiazide-like diuretic presented electrolyte disturbances with hyponatremia or hypokalemia. Moreover, it was demonstrated that syncope and falls were significantly more common among patients receiving a thiazide diuretic than those not treated with these drugs [56].

It should be remembered that hypokalemia can cause severe and life-threatening cardiac arrhythmias. In turn, the individuals with a genetic baseline decrease in the prostaglandin transporter activity (encoded by *SLCO2A1*) are in the risk group for thiazide-induced hyponatremia [57]. Therefore, the patients for whom such treatment was prescribed required regular laboratory monitoring of serum sodium and potassium concentrations.

Comment on Statement 2.5

Several clinical trials have demonstrated that MRA spironolactone lowers BP and reduces proteinuria. In addition, it may delay CKD progression in diabetic or non-diabetic CKD when used on top of an RASi [58,59]. In one trial, spironolactone added to ACEis or ARBs in the CKD setting reduced SBP by 6 mmHg and proteinuria by 40% [59]. Williams et al. demonstrated the efficacy of spironolactone as the 4th drug added following previously used RASi, CCB, and diuretics [60]. The recent meta-analysis showed uncertain effects of aldosterone antagonists added to ACEi or ARB (or both) on the risk of death, major CV events, and kidney failure in patients with proteinuric CKD [59]. Aldosterone antagonists reduce proteinuria and SBP in adults with mild to moderate CKD, but may increase the risk of hyperkaliemia and AKI (particularly when added to ACEi and/or ARB) and gynecomastia [59]. On the other hand, spironolactone may prevent diuretic-induced hypokalemia and is recommended for treating heart failure. Still, careful monitoring of serum potassium is mandatory in CKD. Hyperkaliemia is the critical limitation of the widespread use of aldosterone antagonists in patients with advanced CKD. Due to the risk of hyperkalemia, it should not be started in patients with an eGFR ≤ 45 mL/min/1.73 m^2^ and serum potassium > 4.5 mmol/L [61].

The alternatives to spironolactone include eplerenone and finerenone. Due to less potent BP-lowering properties, eplerenone is not used to treat hypertension. Moreover, the antiproteinuric properties of eplerenone remain less documented when compared with spironolactone (especially in non-diabetic CKD) [59]. It can be used as an off-label therapy for blood pressure reduction in patients with spironolactone intolerance (mainly in males with gynecomastia). Finerenone, a novel selective non-steroidal MRA, reduces the risk of kidney function decline and CV events in adults with CKD associated with type 2 diabetes and is currently studied in non-diabetic CKD [62,63].

The use of β-blockers in CKD, although not as a first-line treatment, is well justified given the significant upregulation of the sympathetic nervous system activity observed in CKD, which increases the risk of CV events and renal disease progression [64]. β-blockers are mainly recommended in treating heart failure, arrhythmia, hypertrophic cardiomyopathy, or coronary heart disease, the comorbidities often seen in patients with CKD [65]. Definitive studies to guide β-blocker prescription in CKD are lacking, but their use is common, mainly due to the high risk of CVD in this setting. It should be remembered that bradycardia is a well-described side effect of this drug group, and it is also a common concern among patients with CKD. Dosing adjustments of β-blockers may be required, and hepatically metabolized agents and those with additional vasodilatory properties (such as carvedilol and nebivolol) are likely to be of particular value [66].

Alfa-blockers (alfa-adrenoceptor antagonists, such as doxazosin) are commonly used to treat resistant hypertension in CKD patients. Their pharmacokinetic profile is independent of kidney function and metabolically neutral [67]. Some small studies showed their efficacy in managing high BP in CKD [68,69]. However, using alpha-blockers is associated with orthostatic hypotension, which should be considered mainly in older patients with CKD. Centrally acting drugs, such as clonidine or methyldopa, are relatively safe in patients with CKD [70]. They can be used in patients with resistant hypertension or when the other BP-lowering medications are contraindicated. In patients with resistant hypertension and normal renal function, clonidine was as effective as spironolactone in lowering BP [71].

## 3. Inhibition of Renin–Angiotensin–Aldosterone Axis

Statement 3.1. We recommend using a pharmacological blockade of the RAS (if not contraindicated) in patients with non-diabetic CKD (G1-G4, A3) (1B). ACEi should be considered a preferred therapeutic option (1B), and ARB may be used in case of ACEi intolerance (2C). Doses of ACEi or ARB should be carefully up-titrated with their tolerance monitoring and reduced accordingly with eGFR decline, if needed.

Statement 3.1.1. We recommend using the following ACEi for nephroprotection: benazepril, ramipril, lisinopril (1B), but it is possible to use other ACEi (2D).

Statement 3.1.2. We recommend using ACEi or ARB in maximum tolerated doses (according to summaries of product characteristics) (2C).

Statement 3.1.3. We suggest that ACEi or ARB for nephroprotection should be accompanied by dietary salt intake restriction (2C).

Statement 3.1.4. We suggest monitoring serum creatinine and potassium concentrations within 7–14 days after initiating or increasing the dose of an ACEi or ARB [expert opinion].

Statement 3.1.5. If serum creatinine concentration increases less than 30% of the baseline after initiating ACEi or ARB or increasing their doses, we suggest continuing an ACEi or ARB. In the case of an increase of more than 30%, we suggest discontinuation of ACEi or ARB and evaluation for renal artery stenosis [expert opinion].

Statement 3.1.6. In case of hyperkalemia (5.0–5.5 mmol/L) after initiating ACEi or ARB or increasing their doses, we suggest using methods that reduce the serum potassium level (preparations that reduce potassium absorption in the gastrointestinal tract, thiazide or loop diuretics, treatment of metabolic acidosis). In case of hyperkalemia (>5.5 mmol/L) after initiating ACEi or ARB or increasing their doses, we suggest the discontinuation of these agents, further monitoring of serum potassium, and the possible resumption of ACEi or ARB treatment at a lower dose in combination with measures to lower serum potassium [expert opinion].

Statement 3.2. We suggest using a pharmacological blockade of the RAS (ACEi or ARB) (if not contraindicated) in patients with non-diabetic CKD (G1–G4, A2) (2C) and CKD (G1–G4, A1) (2D).

Statement 3.3. We recommend not stopping ACEi or ARB in advanced CKD (G4–G5) to increase the glomerular filtration rate or slow its decline (1B).

Statement 3.3.1. Dosing or discontinuing ACEi or ARB in symptomatic hypotension or uncontrolled hyperkalemia despite medical treatment should be considered [expert opinion].

Statement 3.4. We recommend not using a combination of ACEi and ARB (1A).

Statement 3.5. We suggest using a combination of ACEi or ARB and MRA—spironolactone (if not contraindicated, i.e., mainly in patients with a tendency to hyperkalemia—serum potassium concentration > 4.5 mmol/L) in patients with non-diabetic CKD (A2–A3) with persistent albuminuria despite the use of ACEi or ARB. Such treatment should be carried out with frequent monitoring of serum potassium concentration (2D).

Comment on Statement 3.1

This recommendation is strong and based on several RCTs with essential benefits in CKD patients. The Gruppo Italiano di Studi Epidemiologici in Nefologia (GISEN) performed the Ramipril Efficacy In Nephropathy (REIN) study that compared ramipril to placebo to assess the effect of ACEi on CKD progression independent of blood pressure lowering. In the group of 166 patients with a mean GFR of 40.2 mL/min/1.73 m^2^ and proteinuria of 3 g/24 h (REIN-Stratum 2), the treatment was stopped early due to the efficacy of ramipril (5 mg daily) in slowing GFR decline. The monthly decrease in GFR was significantly lower in the ramipril group (0.53 mL/min) than in the placebo group (0.88 mL/min; *p* = 0.03). The composite of doubling of the serum creatinine or ESKD was reached in 18 versus 40 participants (ramipril vs. placebo, *p* = 0.02) [72]. In the REIN Stratum-1 of the study, including 186 patients with mean a GFR of 49.5 mL/min/1.73 m^2^ and proteinuria of >1 to <3 g/24 h, the rate of decline in GFR was not different. Still, ESKD events were less frequent with ramipril (9 cases/99 patients) than with placebo (18 cases/87 patients) [24].

The Angiotensin-Converting Enzyme Inhibitors and Kidney Protection (AIPRI) trial compared ACEi and benazepril to a placebo in patients with mild to moderate CKD (mean GFR—37.1 mL/min/1.73 m^2^), mostly without diabetes, to assess its effect on CKD progression (doubling of serum creatinine or ESKD comprised the primary endpoint). The trial involved 583 patients from 49 centers in Italy, France, and Germany. The patients were randomized to benazepril (10 mg daily) or placebo plus other antihypertensive agents, with a target DBP of less than 90 mmHg. Benazepril caused a 53% reduction in the primary outcome (RR: 0.47; 95% CI, 0.27–0.70). After statistical adjustment for changes in DBP, the risk reduction yielded by benazepril was 38%. The best preservation of renal function was observed in patients with chronic glomerular diseases and proteinuria greater than 1.0 g/24 h [34]. In another analysis of 224 patients with advanced nondiabetic CKD (baseline serum creatinine range: 3.1–5.0 mg/dl and mean proteinuria 1.6 g/24 h), Hou et al. compared the effect of benazepril (20 mg daily) vs. placebo on top of conventional antihypertensive therapy on a composite renal endpoint comprising the doubling of serum creatinine, ESKD, or death. Over a mean follow-up of 3.4 years, the risk of reaching this endpoint was 43% lower in the benazepril group than in the placebo group. Additional benefits of the ACEi therapy included a 52% reduction in proteinuria and a 23% slower rate of GFR decline [73].

In the AASK study, 1094 hypertensive patients of African American descent with CKD (mean baseline eGFR: 45.6 mL/min/1.73 m^2^; mean urinary protein excretion 0.6 g/24 h) were randomized to initial blood-pressure-lowering treatment with metoprolol (50–200 mg daily; n = 441), ramipril (2.5–10 mg daily; n = 446), or amlodipine (5–10 mg daily; n = 217) in a 3 × 2 factorial design. Compared with the metoprolol and amlodipine groups, the ramipril group manifested risk reductions in the clinical composite outcome (decrease from baseline in eGFR by 50% or greater, incident ESKD, or death) of 22% (95% CI, 1–38%; *p* = 0.04) and 38% (95% CI, 14–56%; *p* = 0.004), respectively [44].

No studies demonstrated that, compared with placebo, ARB reduced the risk of ESKD in patients with non-diabetic CKD. However, the evidence must be sufficiently robust to show that ACEi is better than ARB. Despite differences in the mechanism of action, experimental and clinical studies reveal similar improvements in the glomerular hemodynamics of ACEi and ARB. Both drug classes have equivalent effects on the major determinants of CKD progression, namely, blood pressure and proteinuria. The Renoprotection of Optimal Antiproteinuric Doses (ROAD) study directly compared the ACEi benazepril to the ARB losartan in 360 patients with CKD without diabetes and with the mean proteinuria of 1.4–2.0 g/24 h. No differences were found in kidney outcomes between the two classes of RAS-blocking agents [41]. Further, in the systematic review and Bayesian network meta-analysis of 119 RCTs (n = 64,768), ACEi and ARB reduced the odds of kidney failure by 39% and 30% (odds ratios (ORs) of 0.61 (95% CI, 0.47–0.79) and 0.70 (95% CI, 0.52–0.89)), respectively, compared to placebo, and by 35% and 25% (ORs of 0.65 (95% CI, 0.51–0.80) and 0.75 (95% CI, 0.54–0.97)), respectively, compared with other active controls. There were no significant differences between ACEi and ARB in this regard [40]. Many studies have also shown that treatment with ARB reduces albuminuria or proteinuria to an extent comparable to ACEi and that this effect is independent of blood pressure lowering [74].

Comment on Statement 3.1.1

We recommend using ACEi, whose nephroprotective potential has been proven in RCTs, i.e., benazepril, ramipril, and lisinopril [34,72,75]. However, we assume that this protective effect is not due to the specific action of those particular agents; similar mechanisms can be attributed to the entire class of ACEi. Hence, it is correct to use other ACEi preparations than those recommended above.

Comment on Statement 3.1.2

We are convinced that ACEi and ARB should be titrated to the maximum tolerated doses approved by regulatory agencies/respective summaries of product characteristics, mainly because the renal benefits were achieved in trials when high doses were used [44,73]. The benefits from RAS-inhibiting agents administered in low doses are less obvious [74]. Evidence exists that the inhibition of the RAS is a dose-related phenomenon. Enhancing the RAS inhibition by increasing the dosage of ACEi or ARB allows for a more significant decrement of proteinuria and attenuation of tubular injury [76]. Therefore, considering the prognostic impact of proteinuria reduction on the renal outcome, it has been commonly recommended to up-titrate ACEi or ARB to achieve the maximum antiproteinuric effect regardless of blood pressure. This was confirmed in the ROAD study, which was performed to determine whether the titration of ACEi, benazepril, or ARB and losartan to optimal antiproteinuric doses would safely improve the renal outcome in CKD. Three hundred and sixty non-diabetic CKD patients with serum creatinine of 1.5 to 5.0 mg/dl and persistent overt proteinuria of >1.0 g/24 h were assigned to four groups. The patients received a conventional dose of benazepril (10 mg/daily), an individually up-titrated dose of benazepril (median 20 mg/daily; range 10 to 40 mg), a conventional dose of losartan (50 mg/daily), or the individually up-titrated dose of losartan (median 100 mg/daily; range 50 to 200 mg). Compared with conventional dosages, optimal antiproteinuric dosages of benazepril and losartan achieved through up-titration were associated with a 51% and 53% reduction in the primary endpoint risk (*p* = 0.028 and 0.022, respectively), with no expense of additional risk of adverse events [73].

It was also speculated that a more aggressive RAS blockade using a single ACEi or ARB in ultra-high doses (two to four times the maximum dose for hypertension) could reduce further proteinuria and reverse the destructive processes within the kidney [77,78,79]. Some exploratory clinical studies conducted in small populations support these hypotheses. The Supra Maximal Atacand Renal Trial (SMART) was designed to assess the effects of supramaximal dosages of candesartan compared with the highest approved antihypertensive dosage of candesartan in Canada (16 mg/daily at the time the study was initiated) in 269 patients with mixed CKD (eGFR > 30 mL/min) and persistent proteinuria ≥1 g/24 h. The mean difference of the percentage change in proteinuria for patients receiving 128 mg/daily candesartan compared with those receiving 16 mg/daily candesartan was −33.05% (95% CI, −45.70 to −17.44; *p* < 0.0001). The reductions in blood pressure were not different across the treatment groups [80]. In an open-label, randomized study intended to evaluate the long-term renoprotective effects of “standard” (80 mg daily) versus “high” (160 mg daily) doses of ARB telmisartan in biopsy-proven chronic proteinuric non-diabetic nephropathies, a high dose of telmisartan seemed to improve the efficacy of the drug to decrease proteinuria and slow the progression to ESKD [81]. However, as long as such management has not been tested in large clinical trials with long-term follow-ups, doses exceeding the maximal approved by regulatory agencies should not be used. Of note, the trials with maximizing ACEi or ARB or combining them were designed when RAA inhibition remained the key strategy to slow down CKD progression. These strategies are likely to be abandoned with the advent of such new drugs as SGLT2 inhibitors.

Comment on Statement 3.1.3

Hypertension is a frequent finding in CKD patients and is considered, among others, a consequence of sodium sensitivity [82]. As mentioned in this document, reducing dietary sodium intake improves BP control. Such an approach may reduce the need to add antihypertensive medications and/or escalate their doses [29]. No data from adequately designed RCTs exist to evaluate the effect of a low-sodium diet on clinically meaningful renal outcomes in patients with CKD. However, the results of exploratory and observational studies indicate that this may be true. In the Chronic Renal Insufficiency Cohort (CRIC) study, a large observational study carried out in 3757 CKD patients followed for almost seven years, high sodium excretion exceeding 4476 mg/24 h was associated with a higher risk of CKD progression than in the group with low sodium excretion (less than 2686 mg/24 h). This association was independent of other essential variables modifying the CKD progression rate, including RAS blocking agents and other antihypertensive medications [83]. These findings are in agreement with a meta-analysis reporting that RAS inhibitors had an augmented antiproteinuric effect in patients on a low-salt diet. In the pooled analysis of 11 studies with 516 participants and follow-ups ranging between 1 and 6 weeks, an average reduction in sodium intake to less than 92 mmol/d (5.4 g salt) was associated with a 41.9% (95% CI, −56.4 to −27.4%) reduction in urinary albumin excretion in patients on concomitant RAS blockade [84]. It should be emphasized that in RCTs in which the nephroprotective effect of RAS inhibitors was evidenced, patients were advised to follow a low-sodium diet [24,34,72]. The synergistic effect of low sodium intake and RAS inhibition may be due to the enhanced angiotensin-converting enzyme activity and increased angiotensin II type 1 receptor density in renal tissue triggered by a high salt intake counteracting the effect of RAS blocking agents on glomerular hemodynamics and proteinuria [85].

Comment on Statements 3.1.4–3.1.6

ACEi and ARB are potent antihypertensive drugs that counteract the vasoconstrictor effect of angiotensin II. Precisely, they cause more significant dilatation of the efferent than afferent glomerular arterioles, resulting in a decrease in intraglomerular pressure, a transient reduction in glomerular filtration, and a possible increase in serum creatinine shortly after the initiation of the therapy [86]. In addition, RAS blocking agents inhibit the action of aldosterone, which results in a greater tendency to hyperkalemia [87]. This can be potentially dangerous, especially in patients with a markedly impaired glomerular filtration rate, in those with atherosclerosis, in older adults, and in patients taking other drugs or dietary supplements that may raise serum potassium. Therefore, we included our suggestions for monitoring and dealing with these potential threats. Our statement is an expert opinion only because no controlled trials exist. It follows the statements from the latest Kidney Disease—Improving Global Outcomes (KDIGO) recommendations for treating hypertension and managing hyperkalemia [88,89]. It is also worth to mention ACEi and ARB can be used in patients with single kidney, possibly with more careful safety measures. All other statements on nephroprotection from this document apply to patients with single kidney.

Comment on Statement 3.2

There are no data from adequately designed RCTs to evaluate the effect of RAS blockade on renal outcomes in patients with non-diabetic CKD with normal to mildly (A1 category) and moderately (A2 category) increased albuminuria. The rationale for using these drugs in CKD A2 patients is derived mainly from a secondary analysis of the Telmisartan Randomised Assessment Study in ACE-Intolerant Subjects With Cardiovascular Disease (TRANSCEND) study. This was a large trial in adults intolerant to ACEi with atherosclerotic vascular disease, but not severely increased albuminuria. Exploratory analyses of subgroups showed that the ARB telmisartan tended to reduce the composite outcome of dialysis or doubling serum creatinine in those with moderately increased albuminuria (CKD A2) or an estimated GFR below 60 mL/min/1.73 m^2^ [90]. Furthermore, Cinotti and Zucchelli examined the impact of the ACEi lisinopril (10 mg) on the progression of kidney disease (GFR was measured by inulin clearance) in 131 patients with non-diabetic CKD and baseline creatinine clearance between 20 and 50 mL/min over 22.5-month follow-up in a prospective, randomized, open-label trial involving 16 Italian renal centers. The mean daily proteinuria at baseline equaled 506 mg and included both A2 and A3 categories of CKD patients; in this trial, the progression to dialysis or ESKD was reduced with lisinopril by 66% (HR: 0.34; 95% CI: 0.01–7.92) compared to antihypertensive therapy without ACEi [75]. Patients with CKD and normal or mildly increased albuminuria (CKD A1) have a relatively low risk of CKD progression. The protective effect of RAS-blocking agents in these patients may be extrapolated from the experience in diabetic CKD. In the pooled analysis of sixteen trials (7603 normoalbuminuric patients with diabetes), ACEi significantly reduced the onset of albuminuria compared to placebo (six trials, 3840 patients; RR 0.60; 95% CI, 0.43 to 0.84) and to calcium antagonists (four trials, 1210 patients; RR 0.58; 95% CI, 0.40 to 0.84) [91]. Given observational and experimental studies demonstrating the non-hemodynamic beneficial effects of ACEi and ARB, such as the attenuation of local inflammation and fibrosis, it is reasonable to expect that RAS blockade may be an effective therapeutic option in normoalbuminuric non-diabetic CKD patients [92,93]. Although cardiovascular protection is beyond the scope of this position statement, it is worth mentioning that treatment with the ACEi ramipril reduced the risk for all-cause mortality in non-diabetic CKD patients with normal-to-moderately increased albuminuria (A1–A2), as was evidenced in the HOPE study [36].

Comment on Statement 3.3

A small observational study showing improved GFR after stopping RAS-blocking agents led to the hypothesis that continuing these drugs in patients with advanced CKD might accelerate the need for kidney replacement therapy [94]. Recent large, real-world observational studies from Sweden and the USA have yielded contradictory results, pointing out that discontinuing ACEi or ARB may increase mortality risk and major adverse cardiovascular events without precise renal benefits [95,96]. The randomized, open-label STOP-ACEi trial was designed to determine whether ACEi or ARB discontinuation could slow CKD progression in patients with stage 4–5 CKD. Four hundred and eleven patients with diabetic and non-diabetic CKD were randomly assigned to discontinue or continue RAS inhibitors. At three years, the least-squares mean (±SE) eGFR was 12.6 ± 0.7 mL/min/1.73 m^2^ in the discontinuation group and 13.3 ± 0.6 mL/min/1.73 m^2^ in the continuation group (difference, −0.7; 95% CI, −2.5 to 1.0; *p* = 0.42). ESKD, or the initiation of renal replacement therapy, occurred in 128 patients (62%) in the discontinuation group and 115 patients (56%) in the continuation group [97]. Despite some apparent limitations (open-label nature, no dosing information), the STOP-ACEi trial evidenced that discontinuing RAS inhibitors in patients with advanced CKD does not improve kidney function (although adverse cardiovascular effects were not observed). The decision to continue or discontinue RAS inhibitors should be made in the context of the individual patient’s clinical presentation, blood pressure control, and treatment tolerability.

Comment on Statement 3.4

Combination therapy with ACEi and ARB concerning kidney protection has been studied extensively for years. Several studies have investigated dual RAS blockade in non-diabetic or mixed cohorts of CKD patients and documented a more significant antiproteinuric effect of combined therapy with ACEi and ARB than monotherapy with either drug group [98,99]. Despite that, primary RCTs not only evidenced that dual therapy does not improve renal outcome, but also noticed that such a combination may carry an increased risk of serious complications such as hypotension, acute kidney injury, and high serum potassium. In the Ongoing Telmisartan Alone and Combination with Ramipril Global Endpoint Trial (ONTARGET), 25,620 participants were randomly assigned to the ACEi ramipril 10 mg daily (n = 8576), the ARB telmisartan 80 mg daily (n = 8542), or to a combination of both drugs (n = 8502; median follow-up was 56 months), and renal function and proteinuria were measured. The primary renal outcome was a composite of dialysis, doubling of serum creatinine, and death. The number of events for the composite primary outcome was similar for telmisartan (n = 1147 (13.4%)) and ramipril (1150 (13.5%); hazard ratio (HR) 1.00, 95% CI 0.92–1.09), but was increased with combination therapy (1233 (14.5%); HR 1.09, 1.01–1.18, *p* = 0.037). The secondary renal outcome, dialysis or doubling of serum creatinine, was similar with telmisartan (189 (2.21%)) and ramipril (174 (2.03%); HR 1.09, 0.89–1.34) and more frequent with combination therapy (212 (2.49%): HR 1.24, 1.01–1.51, *p* = 0.038). The estimated GFR declined the least with ramipril compared with telmisartan (−2.82 (SD 17.2) mL/min/1.73 m^2^ vs. −4.12 (17.4), *p* < 0.0001) or combination therapy (−6.11 (17.9) mL/min/1.73 m^2^, *p* < 0.0001). The increase in urinary albumin excretion was less with telmisartan (*p* = 0.004) or combination therapy (*p* = 0.001) than with ramipril. Although combination therapy reduced proteinuria to a greater extent than monotherapy, it worsened other renal outcomes and increased the rate of adverse effects [100]. Given the high risk of serious complications confirmed by the ONTARGET findings, dual RAS blockade with ACEi and ARB should not be used [88].

Comment on Statement 3.5

As discussed in this document, steroidal mineralocorticoid antagonists (MRA) reduce BP in CKD patients with resistant hypertension [60]. It is also known that mineralocorticoid receptor activation propagates kidney injury: inflammation, fibrosis, and CKD progression [101]. Therefore, regardless of its hypotensive effect, MRA may be an attractive adjunct to nephroprotective therapy. Several studies demonstrated beneficial effects on urinary albumin excretion in non-diabetic CKD by adding the MRA spironolactone to ACEi or ARB therapy [102,103]. However, the potentially beneficial effects on renal outcomes were confounded by an increased risk of hyperkaliemia, a factor limiting prescribing of the steroidal MRA in CKD [58]. This is why the widespread use of such treatment has not been adopted, and the beneficial effect of spironolactone on long-term renal outcomes has yet to be proven. As mentioned about BP lowering, the alternative agents to spironolactone are eplerenone and finerenone. The antiproteinuric properties of eplerenone were less documented than for spironolactone, and so far, it is only registered for the treatment of patients with heart failure [104]. In response to concerns related to hyperkalemia, several new selective nonsteroidal MRAs, including finerenone, were developed. Recently, in a prespecified, pooled individual-level analysis from two randomized trials, Finerenone in Reducing Kidney Failure and Disease Progression in Diabetic Kidney Disease (FIDELIO-DKD) and Finerenone Reduces Risk of Incident Heart Failure in Patients With Chronic Kidney Disease and Type 2 Diabetes (FIGARO-DKD), a reduction in kidney failure outcome with finerenone on the top of standard care with RAS blocking agents was evidenced in patients with type-2 diabetes and albuminuria [105]. Although FIDELIO-DKD and FIGARO-DKD only involved people with diabetes, we believe that MR activation and the associated inflammation and fibrosis may also be relevant in the pathogenesis of nondiabetic kidney disease, and MR antagonism may be an effective therapeutic option in these patients [101]. Therefore, the authors suggest using the steroidal MRA, spironolactone, in nondiabetic patients with persistent albuminuria despite using ACEi or ARB and without the tendency to hyperkalemia (i.e., serum potassium concentration ≤ 4.5 mmol/L). A recently initiated major clinical trial, A Trial to Learn How Well Finerenone Works and How Safe it is in Adult Participants With Non-Diabetic Chronic Kidney Disease (FIND-CKD), will examine finerenone (currently only approved for people with diabetes) on top of ACEi or ARB treatment in nondiabetic CKD [101].

## 4. Sodium–Glucose Co-Transporter Type 1 Inhibitors

Statement 4.1. We recommend using SGLT2 inhibitors in patients with non-diabetic CKD and eGFR < 60 mL/min/1.73 m^2^ to prevent or slow down the progression of CKD (1A). We recommend using SGLT2i with proven efficacy in non-diabetic CKD (dapagliflozin, empagliflozin) (1A).

Statement 4.2. SGLT2i should not be started in patients with eGFR < 25 mL/min/1.73 m^2^ (for dapagliflozin) or <20 mL/min/1.73 m^2^ (for empagliflozin) (1A). Both drugs might be continued in patients with eGFR below respective thresholds until dialysis or renal transplantation, if tolerated, for renal and cardiovascular benefit (2B).

Statement 4.3. The efficacy of SGLT2i may differ depending on the etiology of CKD [expert opinion]. In some etiologies of CKD, the safety and efficacy of SGLT2i therapy remain unknown (1A).

Statement 4.4. We recommend adding SGLT2i to ACEi or ARB as the first-line nephroprotective agent whenever possible, especially in patients with increased albuminuria (1A). In case of contraindications or intolerance to ACEi/ARB, using these drugs is not a prerequisite to starting SGLT2i therapy (2B).

Statement 4.5. There is no clear evidence to recommend the additional measurements of serum creatinine concentration, eGFR, and serum sodium and potassium concentration after the commencement of treatment with SGLT2i; the monitoring of these parameters should follow standard guidelines [expert opinion].

Comment on Statement 4.1

Two pivotal trials have proven the efficacy of SGLT2i in patients with non-diabetic CKD. The Dapagliflozin and Prevention of Adverse Outcomes in Chronic Kidney Disease (DAPA-CKD) study recruited 4304 patients, of whom 32.5% were non-diabetic. The mean eGFR in this trial equaled 43.1 mL/min/1.73 m^2^, and only 11% had eGFR ≥ 60 mL/min/1.73 m^2^. Seventy-five percent were in CKD stage 3, and the remaining 14% were in CKD stage 4. Median UACR (965 and 934 mg/g) and the percentage of patients with UACR >1000 mg/h (48.7 and 47.9% in dapagliflozin-treated and placebo groups) pointed to a high risk of CKD progression. The DAPA-CKD trial investigators carefully analyzed the underlying etiology of CKD: in 16% of patients, CKD was attributed to ischemic/hypertensive nephropathy; in 6.3%, IgA nephropathy (IgAN); and in another 2.7%, focal/segmental glomerulosclerosis (FSGS). All glomerulopathies were confirmed based on the kidney biopsy results. Interestingly, although more than 67% of the trial patients had type 2 diabetes (T2D), only 58.3% of CKD was attributed to diabetic kidney disease (DKD) (leaving a relatively high number of T2D patients with other kidney diseases identified) [106,107]. The primary composite outcome in this trial was defined as the first occurrence of the following: the permanent decline in eGFR of ≥50%, ESKD (commencement of dialysis, renal transplantation, or permanent reduction in eGFR < 15 mL/min/1.73 m^2^), or death from renal or cardiovascular causes. The key secondary composite outcome was defined as renal events included in the primary composite outcome (i.e., without death from cardiovascular causes). Hospitalization for heart failure or death from cardiovascular causes was analyzed as a composite cardiovascular outcome; all-cause death was also studied. The primary composite outcome was reduced in dapagliflozin-treated patients by 39% compared to placebo; when the composite renal outcome was analyzed, this reduction increased to 44%. Hospitalization for heart failure or death from cardiovascular causes was reduced by 29%, and all-cause mortality by 31%. The study mirrored the spectacular effects of the trial performed earlier in DKD patients, i.e., Canagliflozin and Renal Events in Diabetes with Established Nephropathy Clinical Evaluation (CREDENCE), except for all-cause death, which—although very close to statistical significance—was not reduced by allocation to canagliflozin [108].

Dapagliflozin was equally effective in the diabetic and non-diabetic patients included in the study regarding the primary composite outcome, renal outcome, composite cardiovascular outcome, and all-cause death. All listed benefits in patients without diabetes tended to be greater in non-diabetic patients. It should be emphasized that the benefits of dapagliflozin were independent of age, gender, race/geographic region, baseline estimated eGFR (˂45 vs. ≥45 mL/min/1.73 m^2^), UACR (≤1000 vs. >1000 mg/g), or blood pressure [106,107]. However, the separate analysis performed in patients with CKD stage 4 demonstrated no benefit of dapagliflozin in any of the analyzed outcomes [109].

The second pivotal trial demonstrating the efficacy of SGLT2i in patients with established non-diabetic CKD was the Study of Heart and Kidney Protection With Empagliflozin (EMPA-KIDNEY). This study recruited 6609 patients, out of whom 3040 (less than 50%) were diabetic (and only 2057—31% of the whole study group—were thought to suffer from DKD). In this study, the underlying cause of CKD was even better documented DAPA-CKD since as many as 1862 patients had a prior kidney biopsy (with IgAN as a leading diagnosis—817 patients or 12% of all study samples—followed by FSGS, membranous nephropathy, minimal change disease, and other glomerular diseases). In 22% of the study sample, CKD was attributed to hypertensive/renovascular disease. The definition of primary composite outcome only differed in some details from that defined in the DAPA-CKD trial. It also comprised the first occurrence of the progression of kidney disease or death from cardiovascular causes. Still, the kidney progression was defined as ESKD (dialysis commencement or kidney transplantation), a sustained decrease in the eGFR to less than 10 mL/min/1.73 m^2^ (DAPA-CKD: to less than 15 mL/min/1.73 m^2^), a sustained decline in eGFR of ≥40% (DAPA-CKD: of ≥50%), or death from renal causes. Key secondary endpoints included a composite of hospitalization or death from cardiovascular causes, hospitalization for any reason, or death from any cause; the progression of CKD was also analyzed separately (i.e., primary composite outcome without death from cardiovascular causes). The mean baseline eGFR of 37.5 ± 14.8 mL/min/1.73 m^2^ in the EMPA-KIDNEY trial was the lowest value ever among all large SGLTi trials performed to date. The median UACR equaled 412 mg/g, with an interquartile range between 94 and 1190 mg/g. It is worth emphasizing that 34.2% of patients randomized to empagliflozin and 34.8% of those receiving placebo had eGFR < 30 mL/min/1.73 m^2^—in absolute numbers, it created representative groups of 1131 and 1151 patients, respectively, with CKD stage 4. Taken together, EMPA-CKD is the largest and most representative trial, including patients with established CKD with and without diabetes (to compare, the Effect of Sotagliflozin on Cardiovascular and Renal Events in Participants With Type 2 Diabetes and Moderate Renal Impairment Who Are at Cardiovascular Risk (SCORED) trial, which recruited only diabetic CKD patients randomized 10,584 patients, and the Study of Heart and Renal Protection (SHARP), another landmark trial performed in CKD, recruited 9270 patients) [110,111,112].

The primary composite outcome in EMPA-KIDNEY was reduced by 28% in the empagliflozin group vs. placebo (HR 0.72, 95% CI, 0.64–0.82, *p* < 0.001). Significant risk reduction has also been achieved in empagliflozin-treated patients in the following outcomes: hospitalization for any cause, progression of kidney disease, and ESKD or death from cardiovascular causes. In contrast to DAPA-CKD, all-cause mortality was not reduced in the EMPA-KIDNEY trial; such a reduction was also not observed in the case of hospitalization for heart failure or death from cardiovascular causes.

As in the case of the DAPA-CKD trial, the effect of empagliflozin was independent of the presence/absence of diabetes, although numerically, the impact of a drug on primary composite outcome was more significant in diabetic patients (HR 0.64, 95% CI 0.54–0.77) than in those without T2D (HR 0.82, 95% CI 0.68–0.99). The risk reduction was independent of baseline eGFR, and patients in subgroups with eGFR < 30, ≥30 to ˂45 and ≥45 mL/min/1.73 m^2^ experienced similar benefits. However, this was not true for baseline UACR ranges: the benefit of empagliflozin was noted only in subjects with UACR > 300 mg/g (traditionally defined as ‘macroalbuminuria’ or ‘overt proteinuria’), but not in the two remaining UACR ranges (˂30, ≥30 to ≤300 mg/g).

Both of the discussed pivotal trials that recruited non-diabetic CKD patients treated with empagliflozin or dapagliflozin were included in a landmark meta-analysis published recently and co-authored by several authors of the EMPA-KIDNEY trial (it also included other important cardiovascular outcome trials (CVOT) performed in subjects with a high risk of atherosclerotic CVD and/or in patients with heart failure with reduced/preserved ejection fraction) [113]. It is worth remembering that four trials performed in heart failure (HF) patients (Dapagliflozin and Prevention of Adverse Outcomes in Heart Failure (DAPA-HF), Dapagliflozin Evaluation to Improve the Lives of Patients With Preserved Ejection Fraction Heart Failure (DELIVER), Empagliflozin Outcome Trial in Patients With Chronic Heart Failure and a Reduced Ejection Fraction (EMPEROR-Reduced) and Empagliflozin Outcome Trial in Patients With Chronic Heart Failure With Preserved Ejection Fraction (EMPEROR-Preserved)) also included substantial numbers of patients without diabetes (50–55%), and the mean eGFR in these trials ranged between 61 and 66 mL/min/1.73 m^2^ (i.e., right above the threshold defining CKD stage 3, with substantial percentages of patients with eGFR below 60 mL/min/1.73 m^2^). Hence, although these studies did not intentionally search for CKD patients, their results represented either HF or HF combined with CKD [114,115,116,117,118].

When kidney disease progression was analyzed in non-diabetic patients (i.e., non-diabetic subjects participating in DAPA-HF, DELIVER, EMPEROR-Reduced and EMPEROR-Preserved, DAPA-CKD, and EMPA-KIDNEY), a significant 31% reduction was observed (RR 0.69, 95% CI 0.58–0.69); still, it must be acknowledged that the nephroprotective effect was only achieved in the two latter trials and DAPA-HF (with no impact in the other heart failure trials).

Although cardiovascular protection is beyond the scope of this position statement, it is worth mentioning that the meta-analysis of DAPA-CKD and EMPA-KIDNEY has demonstrated no benefit of SGLT2i on cardiovascular death or hospitalization for heart failure, cardiovascular death, non-cardiovascular death, or all-cause death. However, DAPA-CKD has demonstrated a significant risk reduction in the composite of death from cardiovascular death or hospitalization for heart failure, non-cardiovascular death, and all-cause death. These results were essentially the same in patients with and without diabetes, with a trend towards more benefit in non-diabetic patients [119]. As of today, the DAPA-CKD trial remains the only CKD trial demonstrating better all-cause survival in patients using SGLT2i [119].

Comment on Statement 4.2

The data on the safety and efficacy of SGLT2 inhibitors used in non-diabetic patients with low values of eGFR come from four heart failure trials (EMPEROR-Reduced, EMPEROR-Preserved, DAPA-HF, DELIVER) and two CKD trials (DAPA-CKD and EMPA-KIDNEY). As discussed in the previous section of this document, DAPA-CKD and EMPA-KIDNEY trials proved the nephroprotective efficacy of dapagliflozin and empagliflozin, respectively. Concerning HF trials, empagliflozin in patients with reduced ejection fraction was nephroprotective and decreased the rate of decline in eGFR compared to placebo. The remaining three studies were neutral in this issue (the risk of secondary renal endpoints was essentially the same in patients randomized to placebo vs. empagliflozin or dapagliflozin).

The following eGFR values were defined as exclusion criteria in HF trials: eGFR < 20 mL/min/1.73 m^2^ in the EMPEROR-Reduced and EMPEROR-Preserved Trials, eGFR < 30 mL/min/1.73 m^2^ in the DAPA-HF trial, and eGFR < 25 mL/min/1.73 m^2^ in the DELIVER trial [114,115,116,118]. Since there was no interactions of cardiovascular benefit with eGFR value in the HF trials, it can be concluded that cardiovascular efficacy is independent of baseline eGFR; in fact, trends toward greater benefits could be observed in patients with lower baseline eGFR [114,115,116,117,118,120]. Since the available data suggest that non-diabetic patients benefit from treatment with dapagliflozin or empagliflozin independently from their baseline eGFR and that both drugs are at least neutral (if not beneficial) for preserving kidney function, in our opinion, they can be initiated for cardiovascular benefits until eGFR reaches 20 mL/min/1.73 m^2^ for empagliflozin and 25 mL/min/1.73 m^2^ for dapagliflozin. Both eGFR thresholds are established due to a lack of evidence (definition of the inclusion/exclusion criteria) rather than any potential cardiovascular harm expected below respective values. Hence, they can be safely continued (but not initiated) below these thresholds until dialysis or renal transplantation.

The situation is more complicated regarding renal benefits in very low ranges of eGFR. The DAPA-CKD trial recruited patients with eGFR ≥ 25 mL/min/1.73 m^2^. As can be concluded from the secondary analysis of this trial, most of the benefits (both renal and cardiovascular) were absent in patients with CKD stage 4 (eGFR ≥ 15 to ˂ 30 mL/min/1.73 m^2^, in DAPA-CKD > 25 to ˂ 30 mL/min/1.73 m^2^). However, trends toward some benefits could be observed in patients randomized to dapagliflozin [109]. Interestingly, a separate analysis of non-diabetic patients included in this trial indicated that patients within all eGFR strata (≥45, ≥30, and ˂45 and <30 mL/min/1.73 m^2^) experienced a reduced risk of the primary composite outcome. Concerning baseline UACR, cardiovascular and renal benefits were independent of baseline UACR in non-diabetic CKD patients randomized to dapagliflozin. When the eGFR and UACR values were combined to classify DAPA-CKD patients into moderate–high, high, and very high risk of CKD progression according to the KDIGO risk stratification, there was no interaction between risk category and renal and/or cardiovascular outcome, as well as all-cause death [121]. The EMPA-KIDNEY trial recruited patients with eGFR ≥ 20 mL/min/1.73 m^2^. As mentioned above, the primary composite outcome was significantly reduced in patients treated with empagliflozin across all eGFR strata, i.e., the cardiorenal outcome was independent of eGFR value in this trial. The cardiorenal benefits of treatment with empagliflozin seemed limited to patients with UACR ≥ 30 mg/g [110].

In summary, SGLT2i in non-diabetic patients with CKD stage 4 may not necessarily be less efficient in nephroprotection than in patients with eGFR ≥ 30 mL/min/1.73 m^2^. However, it must be admitted that the available data on this patient group are limited.

Several pathological pathways of chronic renal injury are parallel in patients with non-diabetic CKD and CKD following kidney transplantation. A good pathophysiological background exists to use SGLT2i in these patients for nephroprotective and cardioprotective purposes. Retrospective analysis performed with a propensity score matching approach and comparing renal transplant recipients with T2D using SGLT2i vs. non-users demonstrated essentially all cardiovascular and renal benefits previously reported for T2D patients in prospective randomized trials [122]. However, since large prospective randomized trials on the use of SGLT2i in renal transplant recipients (both diabetic and non-diabetic) have not been published, the use of these drugs in this patient group cannot be recommended [123].

Comment on Statement 4.3

Concerning the underlying cause of CKD in the DAPA-CKD trial, dapagliflozin was most effective in DKD and among non-diabetic patients with biopsy-proven glomerular disease (with an apparent lack of effect in those with ischemic/hypertensive nephropathies). The more detailed analysis demonstrated that in patients with IgAN, the primary composite outcome was reduced by 72%, and the composite renal outcome by an astonishing 77% [124]. A subgroup analysis of patients with focal segmental glomerulosclerosis demonstrated a trend towards renal benefits with the use of dapagliflozin, although it did not reach statistical significance [125]. At the time of submission of this position statement, the sub-analyses of the EMPA-KIDNEY trial concerning the particular CKD etiologies were not yet available.

The analysis of renal outcome to the underlying cause of CKD in patients suffering from CKD other than DKD could include only two trials (in HF trials, the underlying causes of CKD were not identified). A meta-analysis performed by the Nuffield Department of Population Health Renal Studies Group and SGLT2 inhibitor Meta-Analysis Cardio-Renal Trialists’ Consortium demonstrated, based on data from the DAPA-CKD and EMPAR-KIDNEY trials, that SGLT2i slows down the progression of CKD in patients with glomerular disease (RR 0.60, 95% CI: 0.46–0.78) and that a solid trend toward benefit can be observed in the case of ischemic/hypertensive kidney disease (RR 0.70; 95% CI: 0.50–1.00). The benefit was not observed when CKD was secondary to other conditions or the etiology was unknown. Indeed, all four trials that included diabetic patients with CKD (CREDENCE, SCORED, DAPA-CKD, EMPA-KIDNEY analyzed together) demonstrated a clear benefit in people with diabetes (RR 0.60, 95% CI, 0.53–0.69) and in the entire cohort of patients (diabetics and non-diabetics) (RR 0.62; 95% CI, 0.56–0.69). Since one of four trials that included patients with DKD demonstrated no effect (namely, the SCORED trial, in which patients with DKD were randomized to sotagliflozin vs. placebo), it seems that the nephroprotective effect of SGLT2i cannot be considered a class effect (at least in DKD) [111]. These data suggest that dapagliflozin and empagliflozin may not be equally effective in chronic nephropathies of different etiologies [113].

The undiscovered land in nephroprotection using SGLT2i is best characterized by the kidney-related exclusion criteria of two landmark trials discussed herein. Patients with CKD suffering from autosomal-dominant or autosomal-recessive polycystic kidney disease, lupus nephritis, or ANCA-associated vasculitis were not included in the DAPA-CKD trial. The study did not recruit patients with signs of immunological activity of an underlying illness (defined as cytotoxic therapy, immunosuppressive therapy, or other immunotherapy for primary or secondary renal disease within 6 months before enrolment). As we already mentioned, the history of organ transplantation was also an exclusion criterion [106]. Less-restrictive exclusion criteria were applied in the EMPA-KIDNEY trial and were limited to polycystic kidney disease, any intravenous immunosuppression therapy in the last 3 months, or ongoing treatment with >45 mg prednisolone (or equivalent). These criteria seemed to allow the inclusion of patients with a broader spectrum of underlying renal diseases (including lupus nephritis and ANCA- positive small-vessel vasculitis) and with ongoing activity (45 mg of prednisolone is now considered a very high dose; the study did not exclude patients using oral immunosuppressive agents; only 3-month period between completing intravenous immunosuppression and screening into the study was expected). Maintenance dialysis, functioning kidney transplant, or scheduled living donor transplant were contraindications to participating in the trial [110]. These distinct criteria may explain the differences in outcome observed in non-diabetic CKD patients in both trials (i.e., relatively lower reduction of the hazard ratio for primary composite outcome in non-diabetic patients recruited into the EMPA-KIDNEY trial and lack of reduction of all-cause mortality); it seems evident that patients in the EMPA-KIDNEY trial had not only lower eGFR (by ~7 mL/min/1.73 m^2^, which is clinically meaningful), but also might have included patients with immunologically active primary and secondary glomerular diseases. To highlight this issue, we should wait for subsequent subgroup analyses from the EMPA-KIDNEY trial concerning the underlying cause of renal disease and the type of concomitant treatment (other than ACEi/ARB and blood pressure lowering agents).

Comment on Statement 4.4

Since 98.4% of patients in the dapagliflozin arm and 97.9% in the placebo arm were taking ACEi or ARB in the DAPA-CKD trial, with respective numbers equaling 85.7% and 84.6% in the empagliflozin and placebo in the EMPA-KIDNEY trial, it seems evident that current knowledge on the nephroprotective effect of SGLT2i in non-diabetic CKD is almost entirely based on such a dual treatment. ACEi/ARB-intolerant patients were eligible for both discussed trials. Still, the percentage of such patients in DAPA-CKD was so low that the sub-analysis of outcome in ACEi/ARB non-users would be difficult to perform [106]. Hence, the trial documented dapagliflozin’s nephro- and cardioprotective effect as an add-on therapy to the standard of care based on renin–angiotensin–aldosterone axis antagonists. Concerning the EMPA-KIDNEY trial, the number of patients not using the ACEi/ARB treatment (473 of those assigned to empagliflozin and 508 randomized to placebo) would likely allow for such an analysis (not available at the time of submission of this statement) [110]. As of today (currently), the clinical efficacy of dapagliflozin and empagliflozin remains unknown, and such data will likely be available from real-world observational trials. On the other hand, there is no good rationale to suspect that SGLT2i would not be effective in non-diabetic CKD patients not taking ACEi/ARB due to contraindications or intolerance.

Comment on Statement 4.5

Three critical issues need to be discussed concerning the additional assessment of serum creatinine concentration, eGFR, and serum potassium concentration after treatment with SGLT2i. All are related to drug safety. The issue of such an additional assessment has been raised by the so-called ‘acute dip of eGFR’ observed universally across all SGLT2i trials. Such a dip usually does not exceed 4–5 mL/min/1.73 m^2^, on average, during the first few weeks following the commencement of treatment, is followed by a gradual rise in eGFR value and is not accompanied by an increase in serum potassium. Such a short-term reduction of eGFR is medically not relevant. The long-term nephroprotective effect does not depend on the presence or absence of an initial acute dip in eGFR [126]. Moreover, secondary analysis of the DAPA-CKD study documented that long-term dapagliflozin nephroprotective properties in CKD patients with a so-called ‘acute dip of eGFR’ are more pronounced. In this study, those with an acute reduction in eGFR >10% experienced a long-term eGFR decline of −1.58 mL/min/1.73 m^2^ per year compared with −2.44 and −2.48 mL/min/1.73 m^2^ per year among those experiencing a less-pronounced reduction or increase in eGFR, respectively (*p*-interaction = 0.05) [127]. This is because a transient decrease in eGFR is attributed to the beneficial impact of SGLT2i on renal microcirculation and hemodynamics—reduced intraglomerular pressure. The Nuffield Department of Population Health Renal Studies Group and SGLT2 inhibitor Meta-Analysis Cardio-Renal Trialists’ Consortium meta-analysis confirmed the phenomenon known from most SGLT2i trials and previous meta-analyses. Regardless of the indication, baseline eGFR value, or presence/absence of T2D, SGLT2i reduces the risk of AKI or remains neutral in this issue. Considering two pivotal trials in non-diabetic CKD, DAPA-CKD was neutral, and EMPA-KIDNEY reduced the risk of AKI (RR 0.63; 95% CI: 0.42–0.97) [113]. It should be emphasized that the risk of severe hyperkalemia was not increased in patients treated with empagliflozin compared to those randomized to placebo in the EMPA-KIDNEY trial (in fact, it was numerically lower in those receiving empagliflozin) [110]. DAPA-CKD remained neutral on this issue (12% non-significant reduction of the risk of severe hyperkalemia defined as serum potassium level ≥ 6 mmol/L). Of note, a meta-analysis that included three landmark CVOTs (Canagliflozin Cardiovascular Assessment Study (CANVAS), Dapagliflozin Effect on Cardiovascular Events—Thrombolysis in Myocardial Infarction 58 (DECLARE–TIMI 58), Empagliflozin Cardiovascular Outcome Event Trial in Type 2 Diabetes Mellitus Patients (EMPA-REG OUTCOME)), two HF trials (DAPA-HF and EMEROR-Reduced), and two trials performed in advanced CKD (CREDENCE and DAPA-CKD) demonstrated that treatment with SGLT2i reduces the risk of hyperkalemia. More importantly, no increased risk of hyperkalemia was observed in patients with CKD and those with heart failure with reduced ejection fraction on concomitant treatment with potassium-sparing mineralocorticosteroid receptor antagonists [128].

## 5. Treatment of Metabolic Acidosis

Statement 5.1. We recommend measuring venous serum or venous blood bicarbonate concentration in all CKD patients. Metabolic acidosis in patients with CKD should be diagnosed when the venous serum or venous blood bicarbonate concentration is lower than 22 mmol/L (2B).

Statement 5.2. We suggest the administration of sodium bicarbonate in CKD patients with metabolic acidosis to prevent the CKD progression to achieve venous serum or venous blood bicarbonate concentration in the range of 24–28 mmol/L (2B).

Comment on Statement 5.1

The results of several studies showed that metabolic acidosis (non-respiratory acidosis) frequently develops in CKD patients [129,130,131,132,133]. In the CRIC (Chronic Renal Insufficiency Cohort) study, which involved 3939 patients with CKD stages 2–4, metabolic acidosis was found in 17% of patients [129]. Skiba et al.’s study with 500 patients with CKD stages 1–5 showed that metabolic acidosis occurred in 20% of patients [130]. The National Health and Nutrition Examination Survey estimates showed that 18% of CKD stage G3b patients are characterized by metabolic acidosis [131]. It has been shown that the frequency of metabolic acidosis increases with CKD progression [130,131,132] and is exceptionally high in CKD patients with hyperkalemia [133]. However, it was documented that even in patients with CKD, stage 1 metabolic acidosis is not rare. In these patients, the frequency of metabolic acidosis reaches 10% [132].

As a result of metabolic acidosis in CKD, numerous metabolic processes are disturbed, leading to the abnormal function of many systems and organs [134,135]. The prospective observational CRIC study showed that metabolic acidosis contributes to CKD progression. In this study, CKD progression (i.e., eGFR reduction by ≥50% or kidney replacement therapy initiation) in patients with serum bicarbonate concentration < 22 mmol/L was about three times more pronounced than in patients with serum bicarbonate concentration > 26 mmol/L [136]. Moreover, the results of retrospective observational studies in CKD patients documented that in patients with serum bicarbonate concentration < 22 mmol/L, mortality is increased when compared with CKD patients with higher serum bicarbonate concentration [137]. Considering the above-quoted studies showing the high frequency of metabolic acidosis in CKD patients and its unfavorable clinical consequences, we recommend measuring venous serum or venous blood bicarbonate concentration in all CKD patients. Because the frequency of metabolic acidosis increases with CKD progression, it is believed that in patients with CKD stage 4 or 5, the determination of venous serum or venous blood bicarbonate concentration should be repeated at least once a year. On the other hand, in patients with serum or blood bicarbonate concentrations of 19–21.9 mmol/L, it is reasonable to confirm a diagnosis using a second measurement [138]. In the diagnosis of metabolic acidosis in CKD, arterial blood collection is not recommended due to the risk of bleeding, local complications associated with arterial puncture procedure, and to avoid damage to the arteries that may negatively influence the future establishment of arteriovenous fistula.

The threshold value of 22 mmol/L for diagnosing metabolic acidosis in CKD was established based on the above-quoted observational studies showing that CKD patients with plasma or venous blood bicarbonate concentrations below 22 mmol/L are characterized by increased CKD progression and higher mortality [136,137].

Comment on Statement 5.2

The first prospective clinical study on treating metabolic acidosis in CKD patients was conducted by de Brito-Ashurst et al. [139]. This study analyzed the effect of sodium bicarbonate treatment versus no study medication on CKD progression in a 2-year prospective, interventional study involving 134 CKD patients with an eGFR of 15–30 mL/min/1.73 m^2^ and serum bicarbonate concentration of 16–20 mmol/L. The treatment aimed to achieve a serum bicarbonate concentration of ≥23 mmol/L. The mean dose of sodium bicarbonate used in this study was about 1.8 g. Patients treated with sodium bicarbonate showed a less severe reduction of eGFR compared to the no-study medication group (1.9 vs. 5.9 mL/min/1.73 m^2^/year). It was also revealed that fewer patients treated with sodium bicarbonate needed dialysis (4 vs. 22) [139].

These results were confirmed by a prospective randomized study entitled the Use of Bicarbonate in Chronic Renal Insufficiency (UBI) [140]. In this study, 740 CKD patients with eGFR < 60 mL/min/1.73 m^2^ and a serum bicarbonate concentration of 18–24 mmol/L were randomly assigned to sodium bicarbonate therapy versus no study medication. The treatment aimed to achieve a serum bicarbonate concentration in the range of 24–28 mmol/L. The dose of sodium bicarbonate used to achieve this goal of therapy was about 6 g/daily. The observation period equaled 30 months. In patients undergoing sodium bicarbonate treatment, the reduction in eGFR was less than in those with no study medication (1.4 vs. 3.4 mL/min/1.73 m^2^). Seven percent of patients using sodium bicarbonate initiated dialysis, compared to 12% with no study medication (*p* = 0.02). Moreover, the mortality in patients receiving sodium bicarbonate was lower than in those who remained untreated (3 vs. 7%; *p* = 0.005) [140].

Hultin et al., in a metanalysis of 15 clinical trials (2445 participants, median follow-up 12 months), showed that sodium bicarbonate therapy compared with placebo or no study medication slowed down the decline of GFR (SMD: 0.26; 95% CI, 0.13–0.40) and reduced the risk of renal replacement therapy initiation (RR: 0.53; 95% CI, 0.32–0.89) [141]. The results of the above-quoted studies and metanalysis documented that sodium bicarbonate therapy prevents CKD progression [139,140,141].

From the clinical practice point of view, sodium bicarbonate should be given two or three times a day. It is generally accepted that the initial daily dose of sodium bicarbonate should be 1–2 g, and then adjusted to reach the target serum or blood bicarbonate. The use of sodium bicarbonate in high doses (i.e., above 6 g/daily), especially during large meals, may lead to a clinically significant increase in stomach volume (by releasing carbon dioxide), which can result in abdominal discomfort and—in the most serious cases (although extremely rare)—in the rupture of the stomach wall [135]. To prevent this complication, it is recommended to use sodium bicarbonate between meals. Dosing sodium bicarbonate in tablets and capsules seems to be more reasonable than in the form of powder. If sodium bicarbonate powder is used, it should be considered that 1/5 teaspoon contains 1 g of sodium bicarbonate powder [135]. The results of a recent UBI study indicate that the target value for serum or blood bicarbonate should be set at 24–28 mmol/L [140].

The results of the above-quoted interventional studies documented that treating CKD patients with sodium bicarbonate is safe [139,140]. The only potential safety issue that should be briefly discussed includes the potential risk of blood pressure elevation following sodium bicarbonate ingestion (due to its high sodium content). As demonstrated in healthy subjects and patients with hypertension, sodium bicarbonate, unlike sodium chloride, does not increase blood pressure [142]. Husted et al. documented a lack of blood pressure increase following sodium bicarbonate administration in patients with advanced CKD and subjects with normal kidney function. Stable sodium balance during the administration of sodium bicarbonate is attributed to the preserved ability of the kidneys to excrete sodium; in contrast, the administration of sodium chloride led to a positive sodium balance [143]. Moreover, the results of the above-quoted long-term clinical studies and metanalysis conducted in patients with CKD also do not indicate that sodium bicarbonate increases blood pressure [139,140].

The importance of the other pharmacological agents, sodium citrate and veverimer, in treating metabolic acidosis in patients with CKD needs to be documented and requires further clinical studies (for a review, see [135]). Recently, a preliminary analysis of the results of the randomized, double-blind, placebo-controlled study VALOR-CKD (Evaluation of Effect of TRC101 on Progression of Chronic Kidney Disease in Subjects with Metabolic Acidosis) was presented. This study randomized 1480 CKD patients with metabolic acidosis to veverimer or placebo. It was found that treatment with veverimer did not slow down CKD progression. However, in the VALOR-CKD study, there was a lack of a clinically meaningful difference in achieved serum bicarbonate concentrations between the veverimer- and placebo-treated patients, limiting the ability to detect a benefit of such treatment of metabolic acidosis [144].

A typical Western diet with meat and a low amount of fresh fruits and vegetables is acidifying and may worsen metabolic acidosis due to CKD [145,146]. In small randomized clinical studies in CKD patients, it was documented that a diet rich in vegetables and fruits prescribed by a dietician and delivered to patients’ homes free of charge corrected metabolic acidosis [147,148,149]. Such a diet may reduce albuminuria in CKD stage 1 and both albuminuria and CKD progression in patients with CKD stages 3 and 4 [147,148,149].

However, it should be noted that the above-cited clinical studies did not fully assess the risk of hyperkalemia during a diet rich in fresh vegetables and fruits in patients with impaired kidney function. In these studies, only CKD patients in stages 3 and 4 with a low risk of hyperkalemia (i.e., patients without diabetes and patients with serum potassium previous concentrations always <4.6 mmol/L) were included [148,149]. Serum potassium concentration was also closely monitored in these patients during the clinical trial. So far, no studies have been conducted on the safety of a diet with a high content of vegetables and fruits in patients with CKD and metabolic acidosis in the conditions of daily medical practice outside the clinical trials. Therefore currently, we do not recommend such a diet as a routine method of treatment of metabolic acidosis in CKD patients.

## 6. Disease/Diagnosis-Specific Nephroprotection (ADPKD, Fabry Disease, Other Diseases

Statement 6.1. Tolvaptan should be used to mitigate the progression of CKD in patients with ADPKD and high progression risk (1A).

Statement 6.2. Agalsidase alpha and beta (1B) and migalastat (2C) should be used to mitigate the progression of CKD in patients with Fabry disease.

Comment on Statement 6.1

Autosomal dominant polycystic kidney disease is the most common hereditary kidney disease, with a prevalence of 1:1000 to 1:2500 persons. ADPKD is a cause of nearly 5% of cases of end-stage kidney disease requiring dialysis or kidney transplantation worldwide [150]. ADPKD has a progressive course mainly related to the increase in the size and number of renal cysts and the development of arterial hypertension. Therefore, current nephroprotective treatment in patients with ADPKD includes strict blood control based on inhibiting the renin–angiotensin–aldosterone system and supportive therapy [151]. The only available drug that may interfere with the specific mechanisms responsible for cyst growth and proliferation is tolvaptan, an aquaretic drug that is a selective, competitive vasopressin receptor 2 antagonist [152]. The effect of tolvaptan in patients with ADPKD has been investigated in more than 20 clinical trials since 2011 [153]. The safety and efficacy of this drug have been studied in three large trials, namely, Tolvaptan Phase 3 Efficacy and Safety Study in Autosomal Dominant Polycystic Kidney Disease (ADPKD) (TEMPO 3:4), Tolvaptan Extension Study in Participants With ADPKD (TEMPO 4:4), and The Replicating Evidence of Preserved Renal Function: An Investigation of Tolvaptan Safety and Efficacy (REPRISE) [154]. TEMPO 3:4 was a randomized clinical trial that included 1445 patients with a total kidney volume of >750 mL and a creatinine clearance of >60 mL/min, aged 18 to 50, with ADPKD. The patients randomly received either a placebo or tolvaptan for 36 months. The change in total kidney volume, which was a primary outcome of this seminal study, was 9.6% and 18.8% in the tolvaptan and placebo arms, respectively. There were also fewer renal pain episodes reported by the patients receiving tolvaptan. Furthermore, a lower rate of kidney function decline was seen in tolvaptan than in the placebo arm. Patients receiving tolvaptan also showed a significant reduction in albuminuria. TEMPO 4:4 was an open-label extension of TEMPO 3:4, in which the long-term effect of tolvaptan was investigated in 871 patients. The change in the total kidney volume from TEMPO 3:4 to TEMPO 4:4 after 24 months was only numerically smaller in patients receiving tolvaptan than in those receiving placebo. The same applied to the yearly change of eGFR. The REPRISE trial included patients aged 18 to 55 years old with eGFR of 25–65 mL/min/1.73 m^2^ as well as patients 56 to 65 years-old with eGFR of 25–44 mL/min/1.73 m^2^ and an eGFR decline of more than 2 mL/min/1.73 m^2^ per year. The patients received either tolvaptan or a placebo for 12 months. After one year, the mean change in eGFR was −2.34 vs. −3.61 mL/min/1.73 m^2^ in the tolvaptan and placebo groups, respectively. Significant differences were seen in all subgroups, except non-white participants and those older than 55 years and with early CKD. Following these trials, tolvaptan was approved in the EU in 2015 and is currently indicated to slow the progression of cyst development and worsening of kidney function in all adult patients with ADPKD [155].

The patients need to be monitored for possible side effects that require special attention, including polyuria, nocturia, and polydipsia, which may increase the risk of dehydration, hepatotoxicity, and hyperuricemia [154]. Tolvaptan must be used with other nephroprotection measures, including inhibiting the renin–angiotensin–aldosterone system, adequate water drinking and fluid intake, and reduced dietary protein and sodium [153,154]. Its use with SGLT2 inhibitors for a potential synergistic kidney protective effect has yet to be investigated in clinical trials [156]. Several other potential treatments have been studied in clinical trials in patients with ADPKD, including metformin, a signal of proliferation inhibitors (sirolimus and everolimus), somatostatin analogues (octreotide and lanreotide), and tyrosine kinase inhibitors. Still, none of them showed a slower decrease in the rate of kidney function decline compared to placebo, despite the modest positive effect on renal cyst growth seen in several studies.

Comment on Statement 6.2

Fabry disease (Anderson–Fabry disease, ORPHA:324) is an ultra-rare, X-linked lysosomal disorder caused by genetic abnormalities in the gene encoding the enzyme α-galactosidase A [157]. The incidence of the disease has been estimated at one in 40,000–117,000 worldwide. The disease affects both males and females and may present in “classical”, “late-onset”, or “non-classical” forms. Clinical symptoms in the classical variant appear during childhood or adolescence in men and usually later in affected women. The most typical symptoms of Fabry disease include acral neuropathic pain (acroparesthesia) with “pain crisis”, gastrointestinal symptoms, CKD with non-nephrotic proteinuria, and hypertrophic cardiomyopathy, which all significantly shorten life expectancy in affected patients [158]. Therefore, managing this disease includes specific therapy and supportive care for gastrointestinal symptoms, pain, and renal and cardiac function. Currently, the available treatment for Fabry disease includes enzyme replacement therapies with recombinant α-Gal A (agalsidase α and agalsidase β) and pharmacological chaperone oral therapy with migalastat, but further treatment options such as substrate-reducing treatment and gene replacement are used in several ongoing clinical trials [159,160,161]. Analysis of two large Fabry disease registries enabled the long-term follow-up of patients receiving enzyme replacement therapy and comparison of their changes in kidney function with historical cohorts of untreated patients. These analyses showed that the treatment slowed the rate of kidney function decline in these patients, thus proving that the recombinant enzyme therapy is nephroprotective [162,163]. Migalastat is a relatively new drug, but a recent 30-month follow-up of patients with sensitive mutations has shown the long-term stabilization of renal function in patients receiving oral migalastat, similar to patients previously receiving recombinant agalsidase enzyme therapy [164]. However, longer-term follow-ups will be required to assess whether oral chaperone therapy can confer specific nephroprotection in Fabry disease. Notably, most patients with Fabry disease do not develop arterial hypertension. They may even present hypotension that limits the use of effective conventional nephroprotective therapies such as inhibitors of the RAS [157,158]. The most recent Expert Consensus on practical clinical recommendations and guidance for patients with classic Fabry disease includes a statement of the potential expected benefit of the combined treatment with recombinant enzyme or oral chaperone with adjunctive kidney protective therapies such as RAS blockade and SGLT2 inhibitor, but the experts conclude that they were unable to identify any evidence from clinical trials to support this recommendation [165].

## 7. Nephroprotective Treatment with Unproven Significance (Treatment of Renal Anemia, CKD-MBD, Oxidative Stress, Inflammation)

### 7.1. Treatment of Renal Anemia

Statement 7.1. After reviewing the available information on the potential nephroprotective effect of correcting renal anemia, conventional ESA or HIF-PHI are currently not recommended as specific therapies to prevent the progressive loss of renal function in patients with chronic kidney disease (1B).

### 7.2. Control of Mineral Bone Disorder

Statement 7.2. The drugs indicated for altered mineral bone metabolism, including calcimimetics, vitamin D and its analogues, and oral phosphate binders, should not be explicitly used for nephroprotection, but may help prevent and control the complications of CKD-MBD [1C].

### 7.3. Prevention and Treatment of Oxidative Stress and Inflammation

Statement 7.3. Based on the available information, no specific anti-oxidative and anti-inflammatory therapies are recommended for nephroprotection (2C).

Comment on Statement 7.1

Renal anemia caused by a relative deficiency of renal erythropoietin production and impairment of iron metabolism affects almost all patients reaching advanced and end-stage CKD [166]. Anemia has also been recognized as a significant contributor to a highly increased risk of cardiovascular disease in CKD and one of the risk factors for kidney function loss [167,168]. The analysis of the known biological effects of endogenous erythropoietin and the pathogenesis of CKD suggest that the treatment of anemia may slow CKD progression. It has been postulated that the results of erythropoiesis-stimulating agents (ESA) might be related to the protection against tissue hypoxia, prevention of oxidative stress, and tubular atrophy–interstitial fibrosis [167]. Interestingly, this likely pathophysiological relationship has yet to be investigated in large studies with ESAs. Most evidence comes from post hoc analyses and small prospective non-controlled trials [167]. Only one large, randomized, prospective study, namely, the Cardiovascular Risk Reduction by Early Anemia Treatment with Epoetin Beta (CREATE) trial that was designed to specifically investigate whether a complete correction of anemia in patients with stage 3 or 4 CKD may improve cardiovascular outcomes and slow the progression of the disease compared with partial correction of anemia, failed to show any significant benefit of hemoglobin normalization on cardiovascular risk and rate of the renal disease progression. The only advantage of this treatment strategy shown in the CREATE trial was improved general health and physical function [169].

To better understand the potential nephroprotective effect of ESA in renal patients, a meta-analysis of 32 controlled clinical trials was performed [170]. The authors of that meta-analysis concluded that ESAs do not provide clinically significant protection for kidney function in patients with CKD [170]. Recently, several oral hypoxia-inducible factor-prolyl hydroxylase inhibitors (HIF-PHIs) were introduced to treat renal anemia in patients with CKD [171]. The only agent from this class currently available in the European Union is roxadustat. Previous studies indicated that all HIF-PHIs can not only correct anemia with the same efficacy as traditional ESA, but also may decrease serum hepcidin levels and thus modulate iron metabolism increasing iron binding and reducing the need for iron supplementation [172]. Also, these drugs may reduce inflammation and oxidative stress in CKD [173]. Unfortunately, there is currently no clinical evidence of a specific nephroprotective effect of HIF-PHI, but the results of preclinical studies are encouraging and justify further clinical trials [174].

Comment on Statement 7.2

Chronic kidney disease–mineral bone disorder (CKD-MBD) is a term recommended by the KDIGO group of experts to define a broad range of systemic disorders of mineral and bone metabolism caused by CKD. Both the severity and rate of progression of CKD-MBD show a strong association with the increased risk for cardiovascular events, mortality, and progression of CKD to end-stage kidney disease [175]. Several key components of CKD-MBD need to be controlled, which may have important implications for kidney disease progression and other CKD outcomes. They include correcting vitamin D deficit, increased parathyroid hormone (PTH) synthesis leading to secondary hyperparathyroidism, and phosphate retention resulting in hyperphosphatemia [176]. Despite a plethora of research, there are still several unanswered questions regarding the optimal treatment of CKD-MBD, including the optimal serum level of parathyroid hormone for various stages of CKD, optimal vitamin D level, and a choice of a form of vitamin D and its compounds that needs to be administered and the use of oral phosphate-binders for kidney and heart protection in CKD stages 1–5 before the commencement of renal replacement therapy [177].

Parathyroid hormone has been known as a non-classical uremic toxin, and its increased levels may be both cardio- and nephrotoxic [178,179]. PTH serum levels rise in parallel with a loss of eGFR due to the uncontrolled proliferation of parathyroid glands (secondary hyperparathyroidism). Despite that, the KDIGO guideline does not recommend a specific target level of PTH at each stage of CKD. Instead, it underlines a need for regular monitoring of its serum level and the introduction of treatment based on an upward trend rather than a specific high value. The options to correct increased serum PTH are limited in non-dialysis patients since calcimimetics (e.g., cinacalcet hydrochloride, etelcalcetide) are contraindicated in such patients because of an increased risk of symptomatic hypocalcemia and worsening of hyperphosphatemia [180]. Thus, the only option in predialysis patients to control secondary hyperparathyroidism is administering vitamin D or its analogue and controlling phosphate and dietary calcium intake. The second potential advantage of vitamin D in this setting is correcting its deficiency, which is highly prevalent in CKD [181]. Several trials have investigated the role of vitamin D and its analogues on clinical outcomes in CKD. Still, none was specifically designed to study their effect on renal function decline. The meta-analysis conducted in 2011 found that all of the evidence gathered was from low- to moderate-quality observational studies and a few randomized controlled trials. They demonstrated that vitamin D supplementation corrected many biochemical parameters used to monitor CKD-MBD, but failed to improve any clinically meaningful outcomes [182]. Paricalcitol is a synthetic vitamin D analogue with less potency for causing hypercalcemia than vitamin D. This drug is recommended to treat secondary hyperparathyroidism in dialysis and non-dialysis patients with CKD [183]. Despite the initial encouraging results, three subsequent randomized controlled trials with paricalcitol conducted in patients with CKD stages 3 and 4 failed to show any decrease in the rate of progression of CKD or the development of end-stage kidney disease compared to placebo [184]. A meta-analysis of placebo-controlled trials that included 21 studies comprising 1894 patients with CKD and secondary hyperparathyroidism confirmed that although paricalcitol reduced the risk of cardiovascular events in CKD patients, it neither improved cardiac structure nor reduced proteinuria or protected renal function [185].

Hyperphosphatemia is another common late consequence of CKD, and high serum phosphate levels were tightly linked to increased cardiovascular complications and mortality in cross-sectional and observational studies [186]. Therefore, not unexpectedly, the current CKD-MBD guideline recommends maintaining normal serum phosphate levels at all stages of CKD, which is an ambitious goal that is not usually reachable. In contrast to chronic dialysis patients in whom the effective control of hyperphosphatemia requires a combination of dietary phosphate restriction, oral phosphate binders, and long dialysis sessions in non-dialyzed patients with CKD, effective phosphate control may only be achieved by dietary phosphate restriction as the prophylactic use of phosphate binders is not generally recommended due to a lack of clinical evidence [175]. In particular, the evidence that using phosphate binders may retard the progression of CKD is missing [175,187].

Comment on Statement 7.3

Oxidative stress, which is defined as a pro-/antioxidant disbalance, is harmful to kidney cells, which are highly metabolically active. Increased oxidative stress has been well demonstrated in various kidney diseases due to a depletion of antioxidants and increased reactive oxygen species generation [188,189]. This subject attracted significant scientific attention in the two last decades, but despite this, no approved antioxidative stress therapy is currently approved for nephroprotection in patients with CKD. A recent meta-analysis of 19 studies reviewed potential antioxidants and anti-inflammatory compounds that might be used in CKD. Despite a high heterogeneity among the studies, antioxidant therapy generally reduced CKD progression [190]. Among the reviewed drug candidates, only two, pentoxifylline and bardoxolone methyl, demonstrated statistically significant kidney protection. Several other compounds, including statins, allopurinol, omega-3 fatty acids, vitamin E, L-arginine, N-acetylcysteine, propolis, and probucol, showed no effect after an adjustment was made for body mass reduction and heart failure-related blood dilution [190].

#### 7.3.1. Plain Language Summary

Chronic kidney disease (CKD) is a condition in which the kidneys are damaged and cannot filter blood as well as they should. CKD is recognized as a global health problem with a prevalence of one in six adults. CKD has a progressive course and is associated with a significantly increased risk of premature death, mainly due to cardiovascular events (myocardial infarction, stroke). Due to non-specific symptoms, more than nine out of ten people with chronic kidney disease are unaware of their kidney disease. Type 2 diabetes is the main risk factor for CKD, followed by arterial hypertension. Primary kidney diseases such as glomerulopathies or inherited diseases such as polycystic kidney disease are much less common causes of CKD. Although chronic kidney disease leads to irreversible kidney damage, it can be prevented by taking appropriate protective measures before kidney damage occurs or by slowing down its progression if the disease has already been diagnosed. These measures constitute so-called nephroprotection. Nephroprotective strategies include both lifestyle modification and pharmacotherapy. Lifestyle modification is a set of individually developed non-pharmacological activities aimed at introducing a healthy diet, weight control, increasing physical activity and avoiding alcohol abuse and smoking. Pharmacotherapy for nephroprotection mainly involves tight control of blood pressure, plasma lipids, and, in diabetic patients, the use of antidiabetic drugs. In addition to these general recommendations, some kidney diseases, such as autosomal-dominant polycystic kidney disease and Fabry disease, require specific nephroprotective measures, such as the use of an arginine vasopressin V2 receptor antagonist or a recombinant enzyme to replace its deficiency, respectively.

Due to the very low awareness of the detrimental health consequences of CKD, it is important to intensively promote evidence-based recommendations for the diagnosis and treatment of CKD among both healthcare professionals and patients with CKD or those at increased risk of this disease. In particular, there is demand for clinical practice-oriented recommendations for renal diseases not caused by diabetes where new studies have recently brought significant progress in management strategies, adding new classes of drugs to nephroprotective regimens like sodium glucose cotransporter type 2 inhibitors, also known as flozins. This new class of previously solely oral antidiabetic drugs has already shown its nephroprotective efficacy and safety in non-diabetic kidney disease. Therefore, the Polish Society of Nephrology has prepared a position paper on nephroprotective therapy in patients with CKD without diabetes. These guidelines help select the optimal nephroprotection approach and also emphasize the importance of the early detection and treatment of CKD.

#### 7.3.2. Summary

We started working on this statement as there is a gap between current guidelines and practice. The authors of this statement follow the steps of colleagues, cardiologists, and diabetologists, who develop and frequently update their pivotal guidelines and recommendations. As has been stated in recent guidelines issued by the European Society of Hypertension, the European Society of Cardiology, and the joint initiative of the American Diabetes Association and European Association of the Study of Diabetes, patients with hypertension, heart failure, and diabetes should be treated aggressively immediately after their respective diagnoses have been established [45,191,192]. Depending on the diagnosis, combined (dual or even triple) pharmacological treatment should be started, additional agents should be added with no delay, and doses should be titrated to the maximum tolerated (though the individual tolerance should always be kept in mind and strict monitoring to detect adverse effects of therapy should be applied). The renal community does not base practice on studies of quality similar to those in hypertension, heart failure, and diabetes. Nevertheless, it is fair to postulate that most non-diabetic CKD patients need a universal nephroprotective approach and should have their treatment based on the four following pillars: effective treatment of hypertension, use of ACEi or ARB, and, in selected patients, spironolactone, SGLT2 inhibitor (as of today, dapagliflozin and empagliflozin are formally registered in such an indication beyond diabetes), and sodium bicarbonate in patients with serum bicarbonate below 24 mmol/L. It seems that drugs representing the respective groups in patients naïve to treatment should be started sequentially rather than simultaneously, but within a period of up to three months. Blood pressure targets, the significant reduction of albuminuria/proteinuria, and the correction of metabolic acidosis should be achieved within three months; certainly, a longer period is needed to slow down the GFR loss rate. We believe that our statement is the most comprehensive and up-to-date, addressing multiple pathways of nephroprotection in patients with non-diabetic CKD.

## Data Availability

Not applicable.
